# A New 2-Aminospiropyrazolylammonium Cation with Possible Uses in the Topical Areas of Ionic Liquids

**DOI:** 10.3390/molecules29102326

**Published:** 2024-05-15

**Authors:** Lyudmila Kayukova, Anna Vologzhanina

**Affiliations:** 1 Laboratory of Chemistry of Synthetic and Natural Drug Substances, JSC A.B. Bekturov Institute of Chemical Sciences, 106 Shokan Ualikhanov Str., 050010 Almaty, Kazakhstan; 2 X-ray Diffraction Laboratory, A.N. Nesmeyanov Institute of Organoelement Compounds, Russian Academy of Sciences, 28 Vavilov Str., B-334, 119334 Moscow, Russia; vologzhanina@mail.ru

**Keywords:** 2-aminospiropyrazolinium structures, azoniaspiro compounds, ionic liquids, in vitro biological activities, phase-transfer catalysts, structure-forming agents, membranes in the current sources

## Abstract

Based on the fact that 2-aminospiropyrazolinium compounds and structurally related azoniaspiro compounds belong, in a broad sense, to the class of ionic liquids, we have reviewed them and studied their practical applications. To search for possible uses of a new 2-aminospiropyrazolinium compounds, it is necessary to undertake a comparison with the related class of azoniaspiro compounds based on available information. The structures of the well-studied class of azoniaspiro compounds and the related but little-studied class of 2-aminospiropyrazolinium have rigid frameworks, limited conformational freedom, and a salt nature. These properties give them the ability to organize the nearby molecular space and enable the structure-forming ability of azoniaspiro compounds in the synthesis of zeolites, as well as the ability to act as phase-transfer catalysts and have selective biological effects. Additionally, these characteristics enable their ability to act as electrolytes and serve as materials for anion exchange membranes in fuel cells and water electrolyzers. Thus, the well-studied properties of azoniaspiro compounds as phase-transfer catalysts, structure-directing agents, electrolytes, and materials for membranes in power sources would encourage the study of the similar properties of 2-aminospiropyrazolinium compounds, which we have studied in relation to in vitro antitubercular, antidiabetic, and antimicrobial activities.

## 1. Introduction

The well-studied ionic liquid (IL) class of azoniaspiro compounds [1] and the little-studied class of spiropyrazolinium structures, developed by us [2], have a salt nature, rigid frameworks, and limited conformational freedom, in which the center and axis of chirality can be present, and these structures are of interest from the point of view of studying their spatial structure as well as their biological and practically useful properties. Here, it is necessary to present the results obtained on spiropyrazolinium compounds and to search for their possible use as ILs by comparing the available information with the related class of azoniaspiro compounds.

Both classes of compounds have the ability to organize the nearby molecular space, causing their selective biological effect in studies of their biomedical properties; furthermore, as has been proven in practice in this case of azoniaspiro compounds, this determines their structure-forming ability when used as structure-directing agents in the synthesis of zeolites and as phase-transfer catalysts, which increases the rate and selectivity of processes. Moreover, these compounds have the ability to act as electrolytes and serve as materials for anion exchange membranes in fuel cells and water electrolyzers. To understand the main provisions of the areas of ILs use and the latest achievements, we present the state of the art of the areas of interest and pay attention to azoniaspiro compounds. 

Thus, the well-studied properties of azoniaspiro compounds should encourage the study of some similar properties of little-studied, new spiropyrazolinium compounds, which we have studied only in relation to their in vitro antitubercular, antidiabetic, and antimicrobial activities.

## 2. Topical Research Areas Related to Ionic Liquids and the Contribution of Spiroammonium Compounds

### 2.1. Synthesis and Structure of the New Biologically Active 2-Aminospiropyrazolylammonium Salts

In arylsulfochlorination reactions of amidoximes, the formation of a spectrum of products has been noted. Thus, stable aromatic and heteroaromatic O-sulfonyl amidoximes, obtained via the reaction of substituted benzamidoximes and pyridine carboxylic acid amidoximes with aromatic and aliphatic sulfonyl chlorides, have been described. The sulfochlorination of structurally different amidoximes can afford O-sulfonyl amidoximes, ureas, and cyanamides [3,4,5,6].

For the first time, we performed the arylsulfochlorination of β-aminopropioamidoximes with six-membered heterocycles in the β-position (piperidine, morpholine, thiomorpholine, and phenylpiperazine) in the presence of diisopropylethylamine (DIPEA) in chloroform and found that a series of 2-aminospiropyrazolinium salts (with cation 2-amino-1,5-diazaspiro[4.5]dec-1-en-5-ium and the anions arylsulfonates, hexafluorophosphate, chloride) formed. Among the arylsulfochlorination products of β-aminopropioamidoximes, their structural isomers O-sulfoaryl-β-aminopropioamidoximes and other possible products, such as ureas and cyanamides, have not been found [7,8,9,10].

Besides the formation of 2-aminospiropyrazolinium structures in the class of β-aminopropioamidoxime derivatives via the Boulton–Katritzky intramolecular rearrangement mechanism, it also occurs during the hydrolysis of 3-(β-aminoethyl)-5-aryl-1,2,4-oxadiazoles at room temperature (r.t.) in H_2_O, DMF + H_2_O, and during HCl hydrolysis. Under the first two conditions, 2-amino-1,5-diazaspiro[4.5]dec-1-en-5-ium benzoates are formed, while the action of HCl on 3,5-disubstituted 1,2,4-oxadiazoles produced hydrochlorides and 2-amino-1,5-diazaspiro[4.5]dec-1-en-5-ium chloride [11,12].

**Figure 1:**
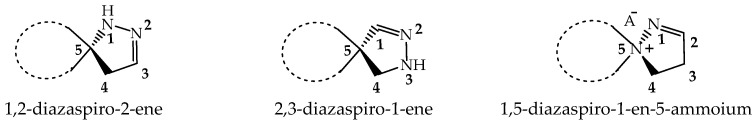
Spiropyrazolines are asymmetric structures with a chirality axis and centers of chirality C-5 carbon atoms in 1,2-diazospiro- and 2,3-diazospiropyrazolines or the ammonium nitrogen atom N(+)-5 in 1,5-diazospiropyrazolinium systems.

Spiropyrazolines, also known as a spirocyclic hydrazine moiety, are rigid asymmetric heterocyclic structures with a chirality axis and with possible centers of chirality of C-5 carbon atoms in the most-studied examples: 1,2-diazospiro- and 2,3-diazospiropyrazolines [13,14] or the ammonium nitrogen atom N(+)-5 in the 1,5-diazospiropyrazolinium systems (Figure 1). There is evidence of tautomerism in the 1,2-diazospiropyrazolines group [15]. 

**Figure 2:**
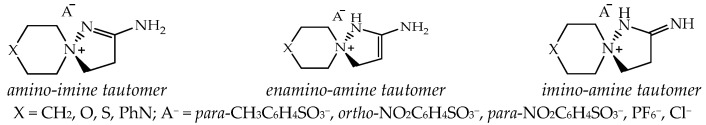
The possible tautomeric forms of 2-aminospiropyrazolinium salts: 2-amino-1,5-diazaspiro-1-en-5-ium; 2-amino-1,5-diazaspiro-2-en-5-ium; 2-imino-1,5-diazaspiro-5-ium, and the synthesized compounds.

According to NMR spectral data and X-ray diffraction data, 2-aminospiropyrazolinium systems exist only in the form of one amino-imine tautomer; no spectral signs of other possible tautomers have been recorded: enamine-amine and imino-amine (Figure 2).

**Scheme 1:**
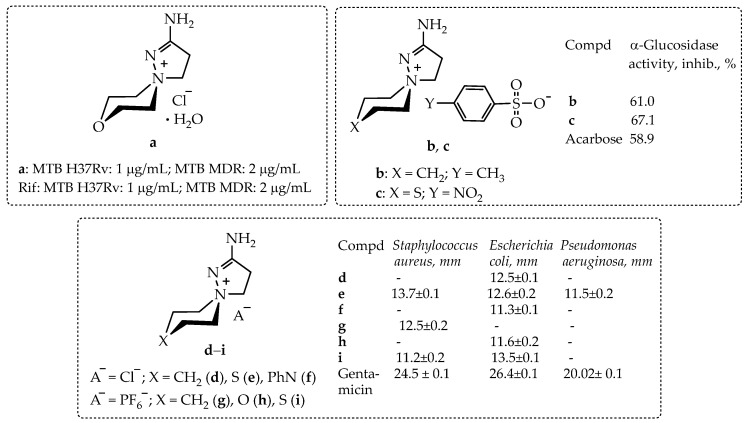
In vitro biological activity of 2-amino-1,5-diazaspiro[4.5]dec-1-en-5-ammonium salts: chloride **a**; arylsulfonates **b**, **c**; chlorides **d**–**f**; hexafluorophosphates **g**–**i**. Data from [10,11,12].

As we discovered, the biological screening of 2-aminospiropyrazolinium containing compounds for a number of in vitro biological activities revealed samples **a**–**i** with different types and levels of activity (Scheme 1).

Thus, compound **a** has an antitubercular activity on sensitive (H37Rv) and multidrug resistant (MDR) strains of *M. tuberculosis*, equal to the activity of the basic antitubercular drug rifampicin; compounds **b** and **c** have antidiabetic α-glucosidase activity exceeding that of the reference drug acarbose [10,12]; compounds **d**–**i** demonstrate pronounced antimicrobial activity on the following bacteria set: *Staphylococcus aureus, Escherichia coli,* and *Pseudomonas aeruginosa*; however, this activity did not reach the activity of the reference drug, gentamicin [11].

Thus, 2-aminospiropyrazolinium compounds, which exhibit high antitubercular and antidiabetic activity, are promising candidates for extended biological tests.

At the same time, 2-aminospiropyrazolinium-containing compounds have not been studied at all in practice in fields beyond a biomedical scope when compared to another related class of compounds, azoniaspiroalkanes. The latter possess numerous practical applications, which are described below. Our interest in the practically useful properties of azoniaspiroalkanes is motivated by a search for other possible ways of using 2-aminospiropyrazolinium compounds developed by us, which, obviously, can be competitive in comparison with azoniaspiroalkanes. 

### 2.2. Green Chemistry and the IL Concepts

#### 2.2.1. The State of the Art

One of the seventeen UN Sustainable Development Goals states the need for global protection of the world for the development and prosperity of people (Goal 12), referring to responsible consumption and production and reducing waste generation through prevention, reduction, recycling, and reuse [16].

This goal essentially includes the concept of “green chemistry”, put forward in 1998. This concept is defined as the use of chemistry to prevent pollution through the application of an appropriate set of products and processes which reduce and, if possible, eliminate the use and formation of hazardous substances [17].

The ACS Green Chemistry Institute (GCI) has been in operation since 2000 within the Office of Sustainability and is part of the Division of Scientific Advancement at the American Chemical Society. The purpose of ACS GCI is to promote and enable the implementation of environmentally friendly and sustainable technologies, chemistry, and engineering on a global scale in chemical plants and society to preserve human health and ecosystems [18].

However, in the United States, the Toxic Substances Control Act of 1976 (TSCA) is a framework law outlining a comprehensive system for controlling toxic substances. It gives the Environmental Protection Agency the authority to regulate the production, processing, distribution, use, and disposal of existing and new chemicals to avoid unreasonable risks to health or the environment and to delay or prohibit production or marketing [19].

Green chemistry is a philosophy of chemical practice with a hazard reduction approach in mind. It can be applied to all aspects of chemistry: raw materials, solvents, processes, products, waste, etc. The resurgence of interest in ILs coincides with the greater awareness of GC [20].

The 12 principles of green chemistry relate to reducing the toxicity of the substances used in synthesis, the formation of the safest possible products and eliminating the generation of waste, atom economy with the participation of all components taken into the processes, the use of energy-efficient processes, catalysts, immediate control of the process with timely prevention of danger in the form of a fire, explosion, etc., as well as the reduction or elimination of auxiliary components of reactions.

New technologies may be adopted if real benefits can be demonstrated. However, the effectiveness of the process must be assessed not only based on the final product but also on the cost in terms of environmental impact and waste toxicity [21].

The chemical industry plays a key role in society through innovative development and the creation of products with numerous beneficial properties. However, at the same time, the toxic and hazardous properties of many solvents, particularly chlorinated hydrocarbons, create a huge threat to the environment in the form of emissions and wastewater pollution. One of the twelve principles of green chemistry is that the use of auxiliary substances, such as solvents, protecting groups, and release agents, should be unnecessary and, if used, should be harmless [22]. It has been recognized that the use of non-traditional solvents, such as ILs, as alternatives to environmentally harmful traditional solvents can reduce the production of waste solvents and, therefore, significantly reduce the negative impact on the environment [23].

The first definition of ionic liquids was as materials composed entirely of ions with melting points, somewhat arbitrarily, below 100 °C [24,25].

The distinctive features of ILs are their immeasurably low vapor pressure and non-flammability. These properties are environmentally friendly for human life. For this reason, they are called green solvents in contrast to traditional volatile and flammable organic solvents [23].

ILs have a set of attractive properties, such as chemical and thermal stability, making them nearly ideal solvents in terms of extraction techniques, and they also have high ionic conductivity and a wide electrochemical potential window.

ILs are a new class of purely ionic salt-like materials that are in a liquid state below 100 °C. This is the existing predominant meaningful definition, leaving significant opportunities for flexibility in perceiving ILs as “designer solvents”.

Therefore, the representative of IL systems is 1-ethyl-3-methylimidazolium chloride aluminum chloride, [emim]Cl^−^AlCl_3_. It is a liquid and retains thermal stability from almost −100 °C to around 200 °C, depending on the molar ratio of [emim]Cl^−^ and A1C1_3_ added to the system. For example, when the molar proportions are equal, the system is a neutral stoichiometric compound, [emim]^+^[AICI_4_]^−^, which melts at a melting point (m.p.) of about 6 °C. The lowest melting point, −96 °C, is achieved when the molar percentages of the system are 35% [emim]Cl^−^ and 65% A1C1_3_.

Chloroaluminate ILs are sensitive to water and air, and harsh conditions are required for their synthesis and utilization [26]. Tetrafluoroborates and hexafluorophosphates in aqueous environments are subject to hydrolysis with the release of toxic gas HF [27].

**Figure 3:**
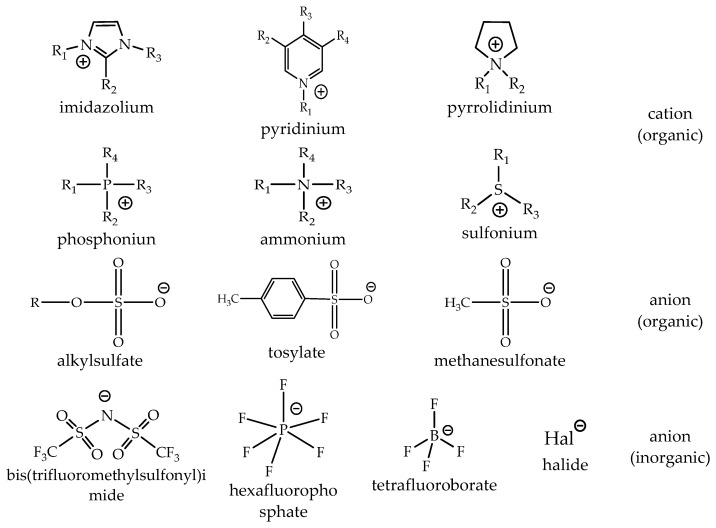
The common ILs and their structures. Reproduced from [28].

The choice of cation has a strong impact on the properties of an ionic liquid and will often define its stability. The chemistry and functionality of ILs are, in general, controlled by the choice of anion. The combination of a wide variety of cations and anions leads to a theoretically possible number of 1018 ILs. However, a realistic number will be magnitudes lower. Today, about 1000 of these compounds are described in the literature, and approximately 300 are commercially available. The typical structures combine organic cations with inorganic or organic anions (Figure 3) [28].

ILs with organic cations such as [emim]+ or [bmim]+ generally have high thermal and chemical stability. ILs are good solvents for a wide range of inorganic, organic, and polymeric materials; moreover, even stones, coal, and almost all organics are highly soluble in ionic liquids.

In organic synthesis, ILs could have a variety of applications in various fields like pharmaceuticals, fine chemicals, biotechnology, medical sciences, nanotechnology, bioremediation, and environmental and nuclear sciences [29]. ILs have played multiple roles such as catalyst, solvent, and catalyst support. In some cases, it was observed that IL enables efficient catalytic reactions in comparison with conventional molecular solvents [30]. Ionic liquids can serve as solvents in reactions using biocatalysis to improve the efficiency and cost-effectiveness and, hence, the sustainability of biocatalytic reactions [31]. Furthermore, ILs serve as extractants of metal ions, organic compounds, and biomolecules [32].

Due to their asymmetry, there are reduced electrostatic forces between the anion and cation in these salts, and the formation of a regular crystalline structure is difficult; therefore, they can remain in a liquid state from temperatures of −20 °C (cholinium and imidazolium ILs) [33] up to elevated temperatures of around 80 °C for bis(fluorosulfonyl)imide (FSI) ILs lithium ion batteries [34].

ILs, which are molten salts at r.t., can meet the latest requirements for lubricants in terms of improving maintenance with reduced emissions and have good properties that prevent the wear of rubbing materials and energy loss.

ILs based on imidazolium dicationic salts can act as lubricants with high temperature stability and can be used as lubricants in severe conditions, such as spacecraft applications with ultra-high vacuums and extreme temperatures (from cryogenic conditions up to 500 °C) at degradation temperature (Td > 415 °C) [35,36,37]. ILs are used as proton carriers in the proton exchange membranes of fuel cells (PEM-FSs) at higher temperatures under non-humidified conditions [38].

An anion exchange membrane (AEM) forms the core of AEM-FCs. AEM-FCs are a promising technology that can solve the high-cost problem of PEM-FCs [39].

Moving toward safer and more environmentally friendly electrolytes, a fluorine-free class of ILs based on dicyanamide and tricyanomethanide anions has been proposed, which have shown promising results when used as the electrolyte for Li metal electrodes [40].

While ILs have attracted great interest in the patent literature, only a limited number of processes (over 50) are known to have been commercialized for large-scale productions [41].

The synthesis of all ionic liquids is a multi-step process. It starts with the ’neutralization’ of a Lewis base, typically alkylimidazoles; trialkylamines, including pyrolles and piperidines, trialkylphopshines, and pyridines are the most frequently used [42]. For the synthesis of protic ILs, the Lewis base (represented as methylimidazole) can be neutralized directly by the addition of a Brönsted acid. This step can occur in conventional organic solvents in which Lewis bases are soluble. Anion metathesis then occurs to the desired ionic liquid. For many ILs, the preparation is strongly associated with the considerations about the required purity of the ILs post-synthesis.

At this technological stage, a water washing stage must be carried out to remove inorganic salts with cations Li^+^, Na^+^, or NH_4_^+^. In the case of a hydrophilic ionic liquid, its extraction will require a large amount of conventional volatile organic solvents that are immiscible with water. Thus, it is obvious that considering ionic liquids as trivial solvents cannot be justified, primarily for economic reasons. And only the unique uses of ILs, which are discussed below (for processing biomass into cellulose, as catalysts, high-temperature lubricants, materials for ion-exchange membranes, pharmaceutical additives, etc.) inspire researchers to create modern materials with useful properties.

Using a design approach to the construction of ionic liquids, it is possible to change their properties, such as viscosity, melting point, density, and hydrophobicity, by variation in the cations and anions and cross the conventional boundary established by the requirement of a ‘true ionic liquid’ existing as a melt at r.t. Considering the basic structural similarity of ‘true ionic liquids’ and all other compounds consisting of ions, one or two of which are organic, the ionic liquids approach can be applied to all of them.

The huge potential and abilities of such liquids, as well as the restrictive definition limiting melting points to below 100 °C, likely steered research away from higher Tm materials in these families of salts that have applications above 100 °C [43].

While most of the ionic liquid community is focused on low melting temperature salts, their solid analogues are likely to offer similar benefits such as low vapor pressure, high chemical and thermal stability, nonflammability, and scope to tailor the properties as per demand [44].

These features can be well utilized in the rapidly emerging field of phase change materials (PCM) for thermal management of electrical devices and most importantly in solar-thermal energy storage [45].

#### 2.2.2. ILs Used in Industry

The size of the global ionic liquids market is expected to be worth around USD 118.7 million by 2033, increasing from USD 47.9 million in 2023 and growing at a CAGR of 9.5% during the forecast period from 2024 to 2033. Due to the increasing use of ILs in solvents, the market is expected to grow substantially over the forecast period. The demand for this product is expected to rise, taking into account growing environmental awareness and green chemistry [46].

The world’s leading companies trading ionic liquids are BASF SE (Ludwigshafen, Germany), TCI America (Portland, OR, USA), SOLVIONIC (Toulouse, France), KOEI CHEMICAL CO. LTD. (Tokyo, Japan), Solvay (99 locations in 61 countries) [47], Scionix (London, UK), and Proionic GmbH (Grambach, Austria) [48].

The properties of ILs have attracted broad global interest in their use as biomass solvents (cellulose, lignin, hemicellulose, chitin, silk fibroin, and wool keratin) [49]. The selective dissolution of biological macromolecules using ILs can now be carried out under unmatched mild conditions without the evaporation of the solvent molecules; ILs can be easily recycled [50,51,52].

The simple, closed process of making the fibers for clothing has no chemical emissions to the atmosphere, providing an alternative to the coal-fired process for making fibers, such as polyester [53,54].

Chevron has implemented ISOALKY Technology to alkylate lower alkanes to octane, using [bmim][Al_2_Et_2_Cl_5_] ionic liquid (bmim = 1-butyl-3-methylimidazolium). It has been used for the dimerization reaction both as the solvent and Lewis acid in the presence of a nickel(II)-containing catalyst, where IL, for easier and safer handling, has replaced conventional hydrofluoric or sulfuric acid catalysts [55].

An example is the assortment of Ionic Liquid Webshop «Proionic», the catalog of which reflects the most popular ionic liquids. So, in the Proionic catalog, out of 17 names of ionic liquids, 13 belong to imidazolium cation derivatives, 2 to pyrrolidine cation derivatives, and 2 items to 2-hydroxyethyl-trimethylammonium. A group of six ionic liquids, consisting of cholinium-L-lysinate and five imidazole-containing ionic liquids intended for processing biomass into cellulose, is separated into a separate group [56].

#### 2.2.3. The Toxic Properties of ILs; Their Screening with Organisms of Different Trophic Levels

A one-sided perspective of ionic liquids as “green” solvents based solely on their non-volatility and non-flammability does not guarantee their overall non-toxicity. Like all chemical products, ionic liquids require a rigorous, comprehensive approach in terms of their impact on human health and the environment. Although ILs can lessen the risk of air pollution due to their low vapor pressure, they have significant solubility [57] as well as high stability [58] in water.

Additionally, the expanding range of IL applications in many fields of the chemical industry has led to a growing threat of contamination to aquatic and terrestrial environments by these compounds [59,60,61].

In addition, the toxicity of ILs should be a focus not only in applications related to biomedical research but also in all other cases where people have to interact with them directly in production processes, from the laboratory to industrial scales. There is a limited number of studies on the toxic properties of ILs compared to the number of studies on their numerous applications. Thus, out of the 8000 publications discovered (in the Web of Science database) devoted to the applied issues of ILs, only 250 (or 3%) studied the toxic properties of ILs [62].

In the last 20 years, the toxicity of ILs towards cells and microorganisms has been heavily investigated, with the main aim of assessing the risks associated with their potential use in industrial applications and developing strategies to design greener ILs [63].

Another important issue is related to the effect of ILs on different organisms. For example, an IL can be toxic to one organism but practically harmless to another; therefore, it is important to analyze the toxic effect of ILs on different organisms, from simpler ones (bacteria or fungi) to more complex ones (plants and animals) [64].

The impact of aromaticity on the toxicity of different cations (pyridinium, piperidinium, pyrrolidinium, and imidazolium) and hydrophobic anions (bis(trifluoromethylsulfonyl)imide [NTf_2_] and hexafluorophosphate [PF_6_]) has been measured [65]. Standard assays that use organisms of different trophic levels, such as bacteria *Vibriofischeri* (Microtox^®^), green microalgae *P. subcapitata,* and cladoceran *D. magna*, enable evaluations of the consistency of structure–activity relationships across different biological targets. The results clearly indicate that the studied ILs can be divided into two groups that present very different dependencies of toxicity in relation to water solubility. One of the groups comprises non-aromatic ILs, which are based on piperidinium and pyrrolidinium. This group presents much lower water solubility and toxicity than aromatic ILs that are based on the imidazolium and pyridinium cations; however, both [PF_6_] and [NTf_2_] anions have little impact on toxicity: [C_3_C_1pyrr_] < [C_3_C_1pip_] < [C_3_C_1im_] < [C_3_C_1pyr_].

When testing imidazolium ILs [C_n_C_1_im][NTf_2_] (n = 1–8), after 15 min of exposure to the luminescent marine bacteria *V. fischeri*, general regularity was demonstrated which, with an increase in the chain length of alkyl radicals C and a subsequent increase in hydrophobicity, increased the toxicity of the ILs [66,67,68,69,70]. Thus, in a row [C_1–8_C_1_im][NTf_2_], the EC_50_ value consistently decreases when moving from n = 1, … to n = 8 from 2362.78 mg·L^−1^ to 6.44 mg·L^−1^.

The reported results suggest the possibility of an IL design with an enhanced hydrophobic character and lower toxicity by eliminating their aromatic nature.

The studies described in the Toxicity Report under the National Toxicology Program [71] evaluate the toxicological potential of selected substances in laboratory animals (two species: rats and mice). The subjects of the research were chosen primarily on the basis of human exposure, level of commercial production, and chemical structure. Three exposure concentrations per IL were used to compare the relative toxicities of the four ILs when administered via drinking water: 1-ethyl-3-methylimidazolium chloride (Emim-Cl), 1-butyl-3-methylimidazolium chloride (Bmim-Cl), 1-butyl-1-methylpyrrolidinium chloride (Bmpy-Cl), and n-butylpyridinium chloride (NBuPy-Cl).

The three-month toxicity studies of the ILs in male and female Sprague Dawley (Hsd: Sprague Dawley^®^ SD^®^) rats and B6C3F1/N mice (n = 10/sex/exposure group) were performed as preclinical studies in accordance with Good Laboratory Practice Regulations.

Overall, the results from this comparative study suggest that Emim-Cl, Bmim-Cl, Bmpy-Cl, and NBuPy-Cl in drinking water have minimal effects on rats and mice at low exposure concentrations (<3 mg/mL). Exposure to higher IL concentrations (≥3 mg/mL) results in lower body weights. The lowest observed effect levels (LOELs) on the exposed male mice were assigned as 10 mg/mL for Emim-Cl, 3 mg/mL for Bmim-Cl, 10 mg/mL for Bmpy-Cl, and 6 mg/mL for NBuPy-Cl. The LOELs for female mice differed slightly. These studies indicate that IL-induced toxicity may be attributable to alkyl chain length and cation type. ILs with longer alkyl chains typically exhibit increased toxicity compared to ILs with shorter alkyl chains. However, the difference in toxicity among cations (imidiazolium vs. pyrrolidinium vs. pyridinium) is less clear and is both sex and endpoint-dependent.

A multidisciplinary study on ILs is emerging, including chemistry, materials science, chemical engineering, and environmental science. More specifically, some important fundamental viewpoints are now different from the original concepts, as insights into the nature of ILs become deeper. For example, the physicochemical properties of ILs are now recognized as ranging broadly from the oft quoted “nonvolatile, non-flammable, and air and water stable” to those that are distinctly volatile, flammable, and unstable [72].

The issue of stability of ILs in any of their uses from laboratory studies to large-scale industrial applications becomes a priority in terms of toxicity both under standard conditions at atmospheric pressure and r.t., without exposure to aggressive factors, and in specific conditions for the using of ILs at high temperatures, aggressive environments, and pressure differences.

Researching the thermal decomposition features of ILs holds paramount importance for the application in battery electrolyte. Imidazole ILs based on nitrates have become the subject of study in terms of their fire and explosion hazards and toxicity of released substances [73,74].

The thermal decomposition peculiarities of 1-allyl-3-methylimidazole nitrate IL were systematically analyzed by means of thermogravimetric analyzer, differential scanning calorimetry, and accelerating rate calorimeter techniques, and the relevant thermodynamic parameters and thermal decomposition models were derived [73]. The results showed that flammable gas, toxic gas, and asphyxiating gas are formed during the pyrolysis of 1-allyl-3-methylimidazole nitrate IL. Besides, [Amim][NO_3_] showed strong potential thermal hazard in non-isothermal adiabatic environment. The heat ΔH generated by adiabatic decomposition was as high as 2330.13 J/g, which presents a high explosive risk. Risk assessments indicate that once thermal runaway occurs, the substance can lead to catastrophic consequences.

The 1-ethyl-3-methylimidazolium bis(trifluoromethylsulfonyl)imide [EMIM][Tf_2_N] has an autoignition temperature of 478 °C. It was observed with the TGA/DSC system that the decomposition of [EMIM][Tf_2_N] was endothermic in a nitrogen atmosphere but exothermic in an air atmosphere [74]. Analysis of the gaseous decomposition products of [EMIM][Tf_2_N] by the TGA-FTIR system indicated that the exothermal effect in the air atmosphere was caused by the autoignition of acetylene, which is one of the gaseous decomposition products [74].

Determination of the degradation products, structure–stability relations, and estimation of the short- and long-term stability were studied for several 1-alkyl-3-methylimidazolium halides. Short-term stability and mechanism of thermal degradation were investigated by a self-developed, innovative thermal analysis single-photon ionization time-of-flight mass spectrometry device with Skimmer coupling. The main decomposition products of the selected ionic liquids were alkyl imidazoles, alkenes, alkyl halides, and hydrogen halides [75].

#### 2.2.4. Creation of the Low-Toxic Ionic Liquids and the Variants of Their Use

The history of ILs dates back to the first generation in 1914 with the synthesis of liquid salts of ethylammonium, which were unstable and sensitive to moisture [76]. The second generation of ILs was marked by the synthesis of a stable 1-ethyl-3-methylimidazolium cation IL, in which the anions were tetrafluoroborate and hexafluorophosphate ([BF_4_] and [PF_6_]) [77]. The enormous possibilities of combinations of organic cations with anions, both organic and inorganic, have led to a variety of IL structures with different physicochemical properties, which can be intelligently designed depending on important applications in fields such as chemistry, energy, pharmaceuticals, etc. Time demands on environmental protection and increased attention to human health are reflected in the development of third-generation ILs. Third-generation ILs are biodegradable cations and anions, and natural compounds containing choline, amino acids, or carbohydrates have been developed for IL production [78].

The main trend in the creation of non-toxic and biodegradable ILs is the use of cationic and anionic fragments from a number of components, which occur in nature and can be metabolized to form non-toxic residues. They are expected to constitute greener replacements for traditional ILs, and their use is associated with applications in the food and pharmaceutical industries [79,80].

Choline, a biologically widespread molecule and an essential micronutrient, is able to degrade completely under aerobic conditions [81]. ‘Bio-ILs’, composed wholly of biomaterials, have been developed in two stages from choline cations and carboxylic acid anions found in nature. In the first stage, a water solution of cholinium iodide was passed through an anion exchange resin (SUPELCO1 AMBERLITE IRA-78) to form [Ch][OH]. [Ch][Ac] was obtained via an ion exchange reaction of an aqueous solution of [Ch][OH] with a slight excess of acetic acid in water. The same method was used to synthesize a series of cholinium ILs with monobasic anions (acetate, glycolate, benzoate, propionate, and tiglate) and acidic anions of dibasic acids (H-succinate, H-malate, H-tartrate, H-maleate, and H-fumarate). After coupling with the choline cation, all new ILs, except for an IL with a H-tartrate anion, were room-temperature ILs (RTILs) with a m.p. below 100 °C. The authors suggested a possible use of these ILs in biomass pretreatment [82].

Cholinium and amino acid-based ionic liquids [AA ILs] occupy a place of their own due to their exceptional biocompatibility stemming from being entirely made of metabolic molecular components. AA is the building unit of proteins and an essential component of our food. Choline (once known as vitamin B_4_) is a metabolite directly synthesized by the human body and is also an essential dietary nutrient [83].

Eighteen promising new cholinium amino acid ionic liquids ([Ch][AA] ILs) have demonstrated biocompatibility and low toxicity toward enzymes and bacteria [84]. Their inhibitory potentials to acetylcholinesterase (EC_50_ values of 2400–3800 µM) are weaker by an approximate order of magnitude than the traditional IL 1-butyl-3-methylimidazolium tetrafluoroborate (EC_50_ value of 330 µM).

The determination of the toxicity of [Ch][AA] ILs and [Ch][AcO] and [Ch][Cl] to representative bacteria (*Escherichia coli*, *Staphylococcus aureus*, *Salmonella enteritidis*, and *Listeria monocytogenes*) has demonstrated that [Ch][AcO] and [Ch][Cl] show lower toxicity towards the test bacteria than [Ch][AA] ILs. However, similar to enzyme inhibitory results, imidazolium-based [Bmim][BF_4_] has been much more toxic than these novel ILs. With relatively few exceptions, [Ch][AA] ILs with lower toxicity have shown higher degradability. Low toxicity and low environmental persistence, combined with their previously demonstrated excellent solvent properties for the selective extraction of lignin, indicate that ILs of this type are highly suitable candidates for large-scale applications.

The Jesus A.R. group studied the dissolving ability of N-acetyl amino acids and N-alkyl cholinium-based ILs when used as co-solvents (0.2–1 mol%) with water in relation to poorly water-soluble drugs, namely sodium diclofenac and paracetamol. In the first case, 20 new biocompatible ionic liquids based on N-alkyl cholinium cations and N-acetyl amino acids anions were prepared [85]. In the second case, the number of ILs was 12; N-alkyl cholinium cations were taken as cations and sulfonate and succinyl alaninate anions as anions [86]. It turned out that these new ILs were able to increase the water solubility of drugs up to four times in pure water and can be considered potential candidates for drug delivery applications.

The transdermal delivery of sparingly soluble drugs is challenging due to the need for a drug carrier. Among the fluid systems for transdermal delivery, attention has been focused on microemulsions (MEs) [87].

The prepared MEs were composed of a hydrophilic IL (choline formate [Ch][For], choline lactate [Ch][Lac], and choline propionate [Ch][Pro]), used as the non-aqueous polar phase, and a surface-active IL (choline oleate [Ch][Ole]) was used as the surfactant in combination with sorbitan laurate in a continuous oil phase [88]. The selected ILs were all biologically active ions.

An in vitro drug permeation study using pig skin showed the significantly enhanced permeation of acyclovir when using a ME. The maximum solubility of ACV in the ILs was 203, 208, and 278 mg/mL for [Ch][For], [Ch][Lac], and [Ch][Pro], respectively, significantly higher when compared with Milli-Q (0.41 mg/mL). Therefore, three [Ch][CA] ILs consisting of choline as the cation and a long-chain (C18) fatty acid as the anion [Ch][Ole] are considered to be safe and biocompatible for further studies relating to transdermal delivery systems.

Cholinium amino acid-based ILs have found applications in an extremely important industry. Chemically recycling poly(ethylene terephthalate) (PET) to monomers is a key strategy in tackling issues arising from persistent plastic pollution in the environment. PET, which is among the most used polymers in modern society, was converted to bis-hydroxyethyl terephthalate (BHET) using ILs based on cholinium salts, including amino acid anions: glycinate ([Ch][Gly]), lysinate ([Ch][Lys]), and alaninate ([Ch][Ala]). The optimal reaction conditions under which a conversion of 85% and a yield of 51% were achieved were obtained via the use of [Ch][Gly] as a catalyst at 150 °C for 6 h [89].

The diversity in structure, the variety in chirality, and the large occurrence of carbohydrates in nature have led to the development of carbohydrate-based ILs, also known as CHILs. They are expected to overcome limitations in aquatic ecotoxicity and poor biodegradability [90].

A systematic study on the influence of different ether groups on the secondary OH groups as well as different configurations on physical properties such as melting point, thermostability, and especially the influence on cell toxicity has been carried out on a series of carbohydrate pyridinium salts based on α- and β-methyl-, β-allyl-, and β-phenyl D-glucopyranose, as well as four 1-deoxypentoses with anions (trifluoromethanesulfonate, tosylate, and mesylate) [91].

**Scheme 2:**
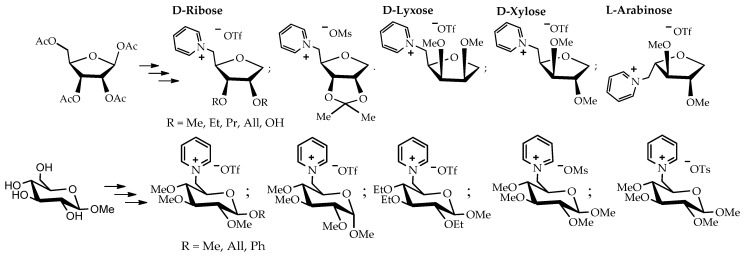
Overview of the 1-deoxy-pentose and β-D-methyl, allyl, and phenyl glucosides as well as the α-D-methyl glucoside-based ILs [91].

The method of introducing a thiophenyl group in the anomeric center followed by reduction with tributyltin hydride was established based on peracetylated D-ribose as the starting compound. As a result of a series of successive stages, a reproducible strategy for the synthesis of new carbohydrate-based pyridinium salts was developed. This strategy was applied to the peracetylated species of D-lyxose, D-xylose, and L-arabinose to synthesize nine new pyridinium salts and seven new pyridinium salts with different glucosides. In the latter case, the starting materials were β-D-methyl, allyl, and phenyl glucosides, as well as α-D-methyl glucoside (Scheme 2).

Thermal analysis showed that 13 of the 16 synthesized derivatives of these new carbohydrate-based pyridinium salts qualify as ionic liquids per the definition, with melting points below 100 °C, with most of them even being room-temperature ionic liquids.

For screening tests on L929 biocompatibility, mouse fibroblasts were used. The results consisted of naturally occurring molecules or their derivatives. Biocompatibility tests proved that these ionic liquids are suitable for biomedical applications as they exhibited much higher viability than common imidazolium or phosphonium-based ILs, which were tested for comparison. 

**Figure 4:**
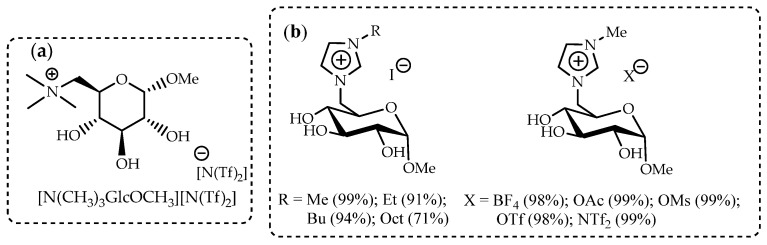
The ILs as the supports for biocatalysts: (**a**) N-(6-deoxy-1-O-methoxy-α-D-glucopyranosyl)-N,N,N-trimethylammonium bis-(trifluoromethylsulfonyl)imide ([N(CH_3_)_3_GlcOCH_3_][N(Tf)_2_]) [92]; (**b**) glucosyl-substituted imidazolium ILs [93].

Catalyst design is considered a key approach to the development of efficient, low-energy, and environmentally friendly processes. Enzymatic acrylation processes are an area where the superior properties of ILs have been demonstrated [92]. Multiwalled carbon nanotubes (MWCNTs) modified by D-glucose-based ionic liquids can be used to form one of the components of the catalytic enzyme complex for lipase B from a *Candida antarctica* (CALB) biocatalyst. The screening of three imidazolium-based ionic liquids, 1-butyl-3-methylimidazolium octylsulphate [bmim][OcSO_4_], 1-butyl-3-methylimidazolium dioctylphosphate [bmim][Oc_2_PO_4_], 1-butyl-3-methylimidazolium bis(trifluoromethylsulfonyl)imide [bmim][N(Tf)_2_], as well as IL based on D-glucose – N-(6-deoxy-1-O-methoxy-α-D-glucopyranosyl)-N,N,N-trimethylammonium bis-(trifluoromethylsulfonyl)imide ([N(CH_3_)_3_GlcOCH_3_][N(Tf)_2_]), showed that the most promising result is obtained in the case of using a D-glucose-based IL (Figure 4a).

Favorable reaction conditions enabled a 99% yield of n-butyl acrylate in 24 h. The developed biocatalytic system could operate for five cycles. The developed method opens a new path to more environmentally friendly, economical, and superior universal catalysts.

A series of novel glucose-based ILs was synthesized in high yields in simple two- or three-step reaction procedures (Figure 4b) [93]. These carbohydrate-based ionic liquids were studied and compared with commercially available imidazolium-based ILs as supports for commercially available biocatalyst Novozym 435 in the acrylation of n-butanol. A direct correlation between the availability of hydroxy groups and the overall activity and enhanced recyclability (up to 10 cycles) of the biocatalyst was found for new glucose-based ILs. All IL-supported Novozym 435 biocatalysts led to similar or overall higher yields of *n*-butyl acrylate than non-IL-supported Novozym 435, with the highest yield being 67 %. Additionally, glucosylimidazolium iodide (R=CH_3_)-supported Novozym 435 had nearly unchanged performance for the tested 10 cycles, lowering to only 62 % from the initial 67 %, marking this IL as the overall best support material for this type of reaction.

Designing drug delivery systems for therapeutic compounds whose receptors are located in the cytosol of cells is challenging due to the fact that the bilayer cell membrane is negatively charged. The newly designed drug delivery systems should assist the mentioned drugs in passing the membrane barriers and achieving their targets. A recent study concentrated on developing novel ILs based on glucose-containing choline, which interacts effectively with the cell membranes. ILs are expected to be non-toxic [94].

The following approximations for the development of ILs based on glucose-containing choline cation and drug substances presented in anionic form have been adopted. Thus, Suksaeree et al. developed a drug-based IL (lidocaine–diclofenac IL), which has a developing effect on controlled drug release. A lidocaine–diclofenac drug IL was prepared via ion pair reactions between hydrochloride salts of lidocaine and sodium salts of diclofenac [95].

Many choline-based ionic liquids have been designed as anti-inflammatory drugs to improve the interaction of anti-inflammatory and antipyretic drugs with biological membranes. Choline-based ILs have increased the solubility of these drugs [96]. Designing new ILs with a choline and hydrogen bond network in their structures can be pursued by combining choline and carbohydrates. The synthesis of the cationic part of these ILs has been reported [97].

In this study, two groups of IL-based drug delivery systems were investigated: (1) systems based on 1-butyl-3-methylimidazolium (BMIM), and (2) systems based on GTA (N-[2-(d-glucopyranosyl)ethyl]-N,N,N-trimethylammonium) cations. The anions included ibuprofen (IBU), aesulfame (ACS), salicylate (SAL), diclofenac (DC), flurbiprofen (FBP), etodolac (ETO), and teophylline (THE).

The most favorable binding free energies on the membrane were observed for paired ILs (BMIM)(ACS) and (GTA)(ACS). These binding free energies were calculated using the molecular mechanics/Poisson–Boltzmann surface area (MM/PBSA) method with MD simulation [98]. Both ionic liquids, (BMIM)(ACS) and (GTA)(ACS), have negative binding energy values towards the membrane (−1645.878 kJ mol^−1^ and −1117.214 kJ mol^−1^, respectively). This indicates that the interactions of both of the ILs with the membrane are thermodynamically very favorable; however, the lower binding energy of (GTA)(ACS) towards the membrane reveals that (GTA)(ACS) can be released from the membrane to the cytosol much easier than (BMIM)(ACS). This result suggests that the (GTA)(ACS) IL can be more effective in terms of drug delivery than (BMIM)(ACS) IL.

### 2.3. Azoniaspiroalkanes with IL Properties Used as Electrolytes

Owing to their distinctive properties, such as their low volatility, high thermal and electrochemical stability, and better ionic conductivity, ILs are extensively used in a variety of energy applications, particularly in the development of green and sustainable energy storage and conversion devices. Suitable ILs have been designed for specific purposes to be used as electrolytes and/or solvents for fuel cells, lithium-ion batteries, supercapacitors (SCs), and solar cells [99,100].

In a continued effort to improve the suitability of ionic liquids in applications operating at raised temperatures, novel spirocyclic ‘azoniaspiro’ salts (with cations derived from five-, six-, seven-, and eight-membered rings) have been prepared and characterized.


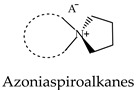


**Scheme 3:**
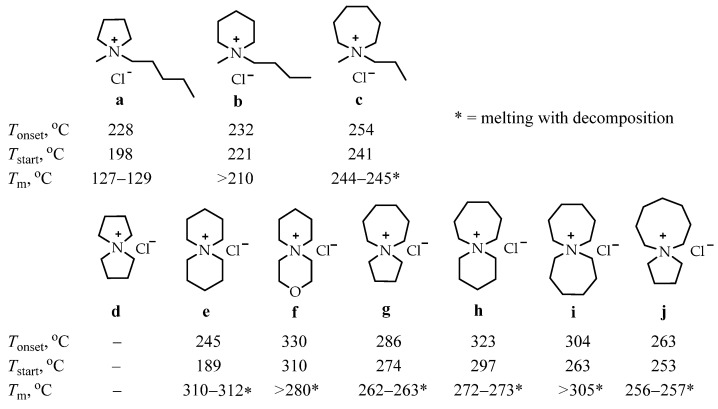
Single-ring (**a**–**c**) and azoniaspiro (**d**–**j**) tetraalkylammonium chloride salts incorporating five-, six-, seven-, and eight-membered saturated rings with their T_onset_, T_start_, and T_m_ temperatures [101].

In order to expand the temperature range in which ILs can operate and study the dependence of IL properties on their structure, a comparative study of the thermal stability of a number of alkyl ammonium chloride salts **a**–**j** was carried out [101]. The inclusion of one or two saturated rings of five, six, seven, or eight atoms into the structure of the studied compounds has been attempted (Scheme 3).

The relative thermal stabilities of the chloride species were compared by employing temperature-ramped thermogravimetric analysis (TGA), and trends were elucidated. The thermal decomposition of ILs usually proceeds through an anionic nucleophilic attack on the α-carbon atom from the ammonium nitrogen of the pendant alkyl chains of the cation.

The authors of the cited work suggested that the inclusion of a second cyclic structure would increase the steric interactions around the ammonium nitrogen center, and α-carbons would be less susceptible to anionic attack. This should provide improved thermal stability to the azoniaspiroammonium chlorides **d**–**j** compared to the tetraalkylammonium chlorides **a**–**c**. The authors’ hypothesis has been confirmed in practice since the salts **e**–**j** show higher T_onset_ and T_start_ values than salts **a**–**c**.

For the single-ring salts **a**–**c**, a slight enhancement in the thermal stability with an increase in the ring size has been observed, from the five-membered pyrrolidinium salt **a** (T_onset_ = 228 °C) to the seven-membered azepanium salt **c** (T_onset_ = 254 °C). [T_onset_ parameter represents a profound overestimate of the long-term thermal stability of IL liquids and related compounds; T_start_ quantifies the first point of appreciable weight loss (d_w_/d_t_ a 0)].

The crystals of azoniaspiro salt **d** were found to be highly hygroscopic, and compound **d** is omitted from the further discussion.

Within the investigated series of the azoniaspiro species **d**–**j**, a significant variation in thermal decomposition behavior was observed—the salts incorporating the five-membered rings **g** and **j** exhibited poorer thermal stabilities than the salts composed of six-, seven-, and eight-membered rings.

The melts of the fully inorganic salts are stable at elevated temperatures but they have high melting points that limit their utility at lower temperatures. It has been proposed that azoniaspiro salts may partially bridge this gap with respect to the stable liquid range.

The thermal properties of azoniaspiro salts represent promising candidates for IL solvent systems, offering the beneficial properties of high thermal stability and a wide electrochemical window. Therefore, analogous to the reputation of ILs as designer solvents, a broad range of structures and properties via modifications to the size and substituents of the two cyclic moieties is clearly presented.

Organic onium cation—5-azoniaspiro-[4.4]-nonane (AS 44), a non-aqueous electrolyte component, has become the subject of inventions [102,103], with reference to a secondary magnesium battery that has a high theoretical capacitive density, long life, and high safety. Secondary magnesium batteries are expected to be introduced into practical use due to their superiority to secondary lithium batteries. A non-aqueous electrolyte consists of an ionic liquid in which the anion is bis((trifluoromethyl)sulfonyl)methanide [N(SO_2_CF_3_)_2_]^−^, and the cation includes Mg^2+^ and an organic onium cation—of 5-azoniaspiro-[4.4]-nonane (AS 44):


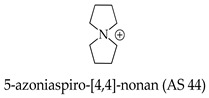


**Figure 5:**
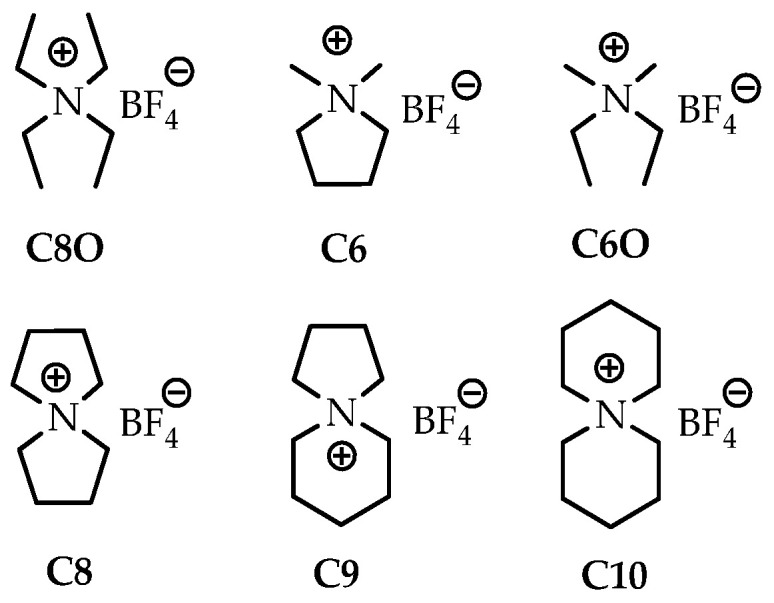
The structure of quarternary ammonium BF_4_ ions. The number after capital C denotes the number of carbon atoms. The designation in the form of the number “0” indicates the absence of cycles in the compound [105].

Quaternary ammonium tetrafluoroborate (QA BF_4_) salts are highly soluble in nonaqueous solvents, conferring high conductivity [104]. Two series of tetrafluoroborates (C80, C6, and C60 and C8, C9, and C10) have been synthesized so that the cations of each series had almost the same molecular weight, with the number of carbon atoms being 6, 8, and 10; however, different structures were achieved in terms of whether it is cyclic or not (Figure 5) [105]. The experiment was performed using acetonitrile on carbon catalysts with a 40 μ thick cellulose separator that was placed between the two electrodes

The effect of the presence of a cyclic structure and its size on the conductivity of the electrolyte has been tested. Cyclic ions have a smaller effective radius than that of corresponding acyclic ions due to fewer degrees of conformational freedom, such as the rotational, bending, and stretching modes. This circumstance leads to the higher capacitances of the cyclic quaternary salts than that of those with their acyclic counterparts. The viscosity (and conductivity) versus the ion size relationship is linear for spiro salts. Thus, an increase in cation size results in higher viscosity and lower conductivity than those in the case of smaller cations.

Electrolytes with high conductivity based on spiro-type quaternary ammonium salts, such as 1,1′-spirobipyrrolidinium tetrafluoroborate (SBP BF_4_) and 4-azoniaspiro-[4.4]-nonane tetrafluoroborate, are being widely investigated for applications in electric double-layer capacitors (EDLCs) [106].

**Figure 6:**
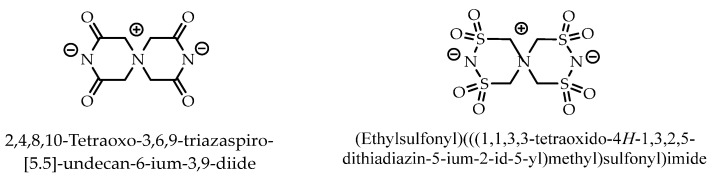
Fluorine-free anions of the azoniaspiro compounds from the “spiros” group. Reproduced with permission from [109]. Copyright 2012 Royal Society of Chemistry.

Currently, lithium-ion batteries (LIBs) are the dominant power sources used in portable electronic devices. However, these batteries have problematic aspects in terms of use due to the fact that they are equipped with fluorinated lithium salts (e.g., LiPF_6_) and organic solvents. Lithium salts are quite expensive for synthesis and unsafe in the presence of moisture; organic solvents have high vapor pressure and are also flammable. Therefore, the design and development of more stable salts and electrolytes are highly desirable [107,108]. In order to mitigate the challenges with the current fluorine-containing electrolytes, the synthesis and evaluation of a new class of fluorine-free lithium and sodium salts based on a pseudo-delocalized concept has been carried out. The representatives of azoniaspiro compounds in the “spiros” group are 2,4,8,10-tetraoxo-3,6,9-triazaspiro-[5.5]-undecan-6-ium-3,9-diide and (ethylsulfonyl)((1,1,3,3-tetraoxido-4H-1,3,2,5-dithiadiazin-5-ium-2-id-5-yl)methyl)sulfonyl)imide (Figure 6) [109].

### 2.4. Green Hydrogen Production and Consumption

#### 2.4.1. The State of the Art

Increasing global warming due to carbon dioxide emissions from burning fossil fuels causes more frequent forest fires, floods, droughts, cyclones, etc., exponentially jeopardizing the global environment [110]. To mitigate the effects of climate change, 154 countries around the world signed the United Nations Framework Convention on Climate Change (UNFCCC) [111]. The UNFCCC takes scientific guidance from the Inter-governmental Panel on Climate Change. It recommends that a global target for greenhouse gas (GHG) emissions (CO_2_, CO, NOx, and SOx) should be set to achieve “net zero” by 2050.

To save Mother Earth, international communities, scientists, and engineers must take appropriate rapid action to switch to new energy technologies, design new approaches to meet future energy demands, and develop technologies that do not entail CO_2_ emissions, such as green technologies [112].

The adoption of hydrogen energy as an alternative to fossil fuels could be a major step towards decarbonizing society and fulfilling the needs of the energy sector [113]. The only route capable of satisfying massive industrial and private energy demands is water electrolysis, which is powered by green electricity produced from sustainable energy sources [114].

In order to continue to advance the use of fuel cells and hydrogen energy technologies, the Fuel Cell and Hydrogen Energy Association (FCHEA) was formed in November 2010 following the merger of two former associations representing different sectors of the industry: the U.S. Fuel Cell Council and the National Hydrogen Association [115].

Electrochemical water splitting, employing renewable power sources, is considered to be a particularly feasible technology for the production of hydrogen without GHG emissions since neither the process of production nor the end products of H_2_ and O_2_ are harmful to the environment; green hydrogen is climate neutral [116]. 

The principle behind fuel cells is the conversion of energy stored in chemical bonds to generate electricity and produce water as waste [117]. The reactions in an alkaline anion exchange fuel cell operating on hydrogen fuel involve the production of energy in the form of electric current via the interaction of hydrogen gas with hydroxyl anions at the anode and the conversion of water into hydroxide anions in the reaction with oxygen at the cathode [118]:

The fuel cell reactions for a hydrogen-fuel alkaline membrane of the fuel cell are described below:

Anode: 2H_2_ + 4OH^−^ → 4H_2_O + 4e^−^

Cathode: O_2_ + 2H_2_O + 4e^−^ → 4OH^−^

Overall: 2H_2_ + 1/2O_2_ → 2H_2_O

Water electrolysis uses DC electricity to split water and generate hydrogen and oxygen gas when a direct current is applied to water [119]:

Cathode: 2H_2_O + 2e^−^ → H_2_ + 2OH^−^

Anode: 2OH^−^ → 1/2O_2_ + H_2_O + 2e^−^

Total: 2H_2_O → O_2_ + 2H_2_

Since the 18th century, water electrolysis technologies have been continuously developed, and some of them have been used in industrial applications. Water electrolysis is the most valuable high-intensity technology for the production of green hydrogen. During these developments, four types of water electrolysis technologies have been introduced based on the electrolyte used, operating conditions, and their ionic agents (OH^−^, H^+^, and O_5_^−^): (i) alkaline water electrolysis (A-WE); (ii) anion exchange membrane water electrolysis (AEM-WE); (iii) proton exchange membrane water electrolysis (PEM-WE); and (iv) solid oxide water electrolysis (SO-WE) [120]. 

The AE cell in alkaline water electrolysis (A-WE) consists of two electrodes (an anode and a cathode) immersed in a highly concentrated aqueous alkaline electrolyte consisting of 20 to 30 mass% KOH/NaOH. Alkaline electrolysis operates at lower temperatures, such as 30–80 °C. In traditional AE, the most commonly used anode and cathode materials are low-cost steel or nickel alloy-plated steel materials. The two electrodes are arranged in a zero-gap (or quasi-zero-gap) formation using a thin diaphragm, which enables the separation of the product gases. The diaphragm is permeable to hydroxide ions and water. The major challenges associated with AE are the handing of the corrosive electrolyte and the limited current densities due to moderate OH mobility. Furthermore, the diaphragm does not completely prevent the crossover of gases from one half-cell to the other. A developing approach in alkaline electrolysis is the use of anion exchange membranes (A-EM) made up of polymers with anionic conductivity instead of an asbestos diaphragm [121].

In PEM-WE, water is electrochemically split into hydrogen and oxygen at their respective electrodes: hydrogen at the cathode and oxygen at the anode. PEM-WE is carried out by pumping water to the anode, where it is split into oxygen (O_2_), protons (H^+^), and electrons (e^−^). These protons travel via a proton-conducting membrane to the cathode side. The electrons exit from the anode through the external power circuit, which provides the driving force (cell voltage) for the reaction. At the cathode side, the protons and electrons recombine to produce hydrogen [122].

PEM-WE uses a dense proton exchange membrane and a pure water feed. PEM-WE is considered to be an exceptional method for high-purity hydrogen production in future industrial applications due to its high current density, greater energy efficiency, smaller gas crossover, and wider operating temperatures (20–80 °C). However, the best-performing catalysts are carbon-supported platinum on the cathode and IrO_2_ on the anode side, and the porous transport layers and bipolar plates are titanium-based to avoid corrosion [123].

This type of electrolysis is efficient but relies on electrocatalysts based on scarce elements such as iridium (to catalyze the anodic oxygen evolution reaction (OER)), which is one of the rarest metals on Earth, and platinum (to catalyze the cathodic hydrogen evolution reaction (HER)). In 2011, the EU identified both of these elements as critical raw materials (CRMs) due to their scarcity, cost, and supply risk [124].

In addition, PEM-WEs currently lean on fluorine-based polymers, which have a significant environmental impact due to the emission of fluorocarbon gases at the production stage of tetrafluoroethylene. The phasing-out of fluoropolymers for environmental reasons has been recently proposed, and the European Commission is currently active in restricting their use in the near future [125].

AEM-WE is an emerging technology that combines the advantages of the mentioned technologies—A-WE (low-cost, abundant materials and pure water) and P-EM (moderate temperature and membrane separation—and appears to be an excellent solution in comparison. The current state of the art in AEM-WE requires a future roadmap for the systematic development and commercialization of systems and components [126].

Table 1 presents a comparison of the current state of the main types of electrolyzers: A-WE, PEM-WE, and AEM-WE; the advantages and disadvantages of the most common types of water electrolysis for hydrogen production have been clearly demonstrated [127].

One of the biggest advantages of AEM-WE is the use of low-cost transition metal catalysts instead of traditional noble metal electrocatalysts. AEM-WE is still in its infancy despite irregular research on catalysts and membranes. In order to generate commercially viable hydrogen, AEM-WE technology must be further developed in terms of energy efficiency, membrane stability, stack feasibility, robustness, ion conductivity, and cost reduction.

Anion exchange membrane water electrolyzers (AEM-WEs) and fuel cells (AEM-FCs) are technologies that can transform and utilize renewable resources in the form of green hydrogen (H_2_) energy. This aspect has significantly reduced the costs of the key components (membranes, electrocatalysts, bipolar plates, etc.), as well as offering fast reaction kinetics and fewer corrosion problems.

The growing interest in anion exchange membrane fuel cells (AEM-FCs) has led to the subsequent development of hydroxide ion-stable cationic polymer anion exchange membranes for water electrolysis [128].

In general, ion exchange membranes (IEM) are semi-permeable membranes composed of ionic head groups attached to the polymer matrices [129]. They can be broadly classified as anion exchange membranes (AEMs) and proton exchange membranes (PEMs) depending on the type of ion, which is permitted to cross the membrane layer [130]. By exploiting the selective nature of IEMs, a variety of applications exist both for AEMs and PEMs. Specific to AEMs, research is focused on developing AEMs for high-pH and high-temperature applications such as anion exchange membrane fuel cells (AEM-FCs) and anion exchange membrane water electrolysis (AEM-WE).

AEM head groups have traditionally been quaternary ammonium (QA) ions; however, current research is also investigating other head groups, such as tertiary diamines, phosphonium, sulphonium, and metal cations (Table 2) [131].

#### 2.4.2. Azoniaspiro Compounds Used as Anion Exchange Membrane Components for Water Electrolysis (AEM-WE) and Fuel Cells (AEM-FC)

AEMs with QA can be formed by reacting a polymer containing a benzyl halide (e.g., chlorine) with an amine (e.g., triethylamine) to add an ammonia group and then treating it with alkaline (e.g., potassium hydroxide) to convert the ammonia group to the salt form, which can participate in anion exchange [132,133].

Among the requirements of AEMs, alkaline stability is considered one of the major challenges, limiting the practical applications of AEM-FCs. AEMs based on the use of quaternary alkylammonium (QA) cation have been extensively studied due to their relatively high hydroxide ion conductivity, easy functionalization, and adequate alkaline stability over short-time scales [134]. Anion exchange membranes based on spirocyclic quaternary ammonium cations have been obtained and studied with the help of both experimental measurements and theoretical calculations.

**Figure 7:**
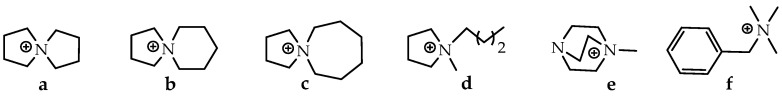
Molecular structures of the studied QAs (**a**–**f**). Reproduced with permission from [134]. Copyright 2011 Royal Society of Chemistry.

Spirocyclic QA cations, including 5-azoniaspiro-[4.4]-nonane [ASN]+ (**a**), 5-azoniaspiro-[4.5]-decane [ASD]+ (**b**), and 5-azoniaspiro-[4.6]-undecane [ASU]+ (**c**) have been synthesized and investigated in terms of their chemical stability in alkaline media at elevated temperatures compared to 1-butyl-1-methylpyrrolidinium [BMPy]+ (**d**), 1-methyl-4-aza-1-azonia-bicyclo-[2.2.2]-octane [MAABCO]+ (**e**), and benzyltrimethylammonium [BTMA]+ (**f**) (Figure 7).

The degradation degree of [ASN]+ in 2M NaOH solution for 168 h has been calculated to be 4.5%, while 2.8% and 6.4%, are observed for [ASD]+ and [ASU]+, respectively. However, the degradation degree observed for nonspirocyclic QA cations [BMPy]+ (9.1%), [BTMA]+ (15.2%), and [MAABCO]+ (14.6%) is significantly higher than that for spirocyclic QA cations. Moreover, most alkali-stable cations [ASD]+ have shown the best alkaline stability in 4 M and 8 M solutions: 3.9% and 31.9%, respectively.

Therefore, the alkaline stability order of the QA cations studied in this work can be ordered as follows: [ASD]+ > [ASN]+ > [ASU]+ > [BMPy]+ > [MAABCO]+ > [BTMA]+.

**Scheme 4:**
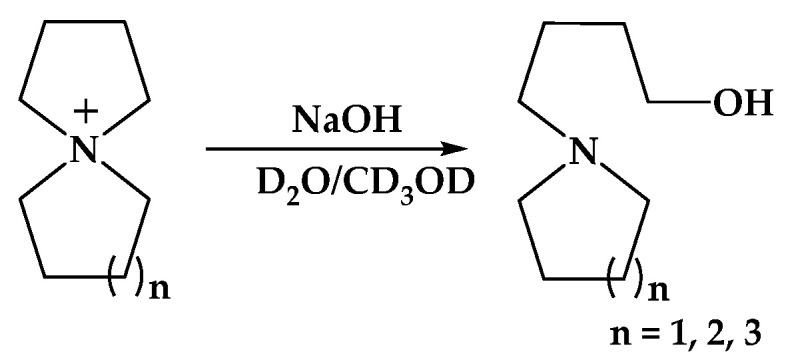
Degradation mechanism of the spirocyclic QAs in the alkaline solution. Reproduced with permission from [134]. Copyright 2011 Royal Society of Chemistry.

In a theoretical study of alkaline hydrolysis, it was found that the Mulliken charge population on the spirocyclic QAs is due to the strong electron-withdrawing effect from an azonia–nitrogen atom, which shows that the neighboring α-C atoms have a positive charge and the faraway β-C atoms show negative charge. Thus, OH^−^ tends to attack the α-C atoms instead of the β-C atoms. To make a parallel comparison and conform the reaction pathway from a computational view, the authors suggested that the ring-opening substitution reaction mainly happens on α-C of the five-membered ring (Scheme 4).

**Scheme 5:**
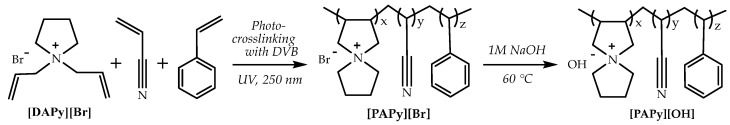
The scheme shows the synthetic route of the anion exchange membrane based on spirocyclic QA [ASN]+. Reproduced with permission from [134]. Copyright 2011 Royal Society of Chemistry.

Based on the alkaline stability of the organic cations studied above, analogous AEMs based on spirocyclic QA have been obtained via the photo-crosslinking of N,N-diallylpyrrolidinium bromide ([DAPy][Br]), N,N-diallylpiperidinium bromide, or N,N-diallylhexamethyleneiminium bromide([DAPi][Br] or [DAHM][Br]) with styrene and acrylonitrile, using benzoin ethyl ether as photoinitiator and divinylbenzene (DVB) as a cross-linking agent via irradiation with UV light with a 250 nm wave length. The prepared polymeric membranes ([PAPy][Br], [PAPi][Br], and [PAHM][Br], respectively) were then immersed in the alkaline solution to convert the membranes from the Br^−^ to OH~ form. AEMs based on the spirocyclic QA cation are free-standing, transparent, and could be very easily cut into appropriate sizes (Scheme 5 for [DAPy][Br]).

The TGA curves of the resulting polymeric membranes based on the spirocyclic QA cation ([ASN]+, [ASD]+, [ASU]+) were recorded under nitrogen atmospheres with temperatures ranging from 30 to 800 °C. The weight loss for [PAHM][OH] at 200 °C was 3.3%, and a less than 2% weight loss was observed below 200 °C for [PAPy][OH] and [PAPi][OH]. Weight loss attributed to polymer degradation begins at 350 °C. [PAPi][OH] was especially thermally stable, with a weight loss of less than 2%; it has good thermal stability and qualifies for AEM applications.

Measuring the conductivity of AEMs based on a spirocyclic QA cation in an alkaline medium was evaluated in a 2M-saturated NaOH solution at various temperatures over a period of time. The conductivities of the membranes increased with increasing temperature. Among these AEMs, [PAPi][OH] showed a loss of conductivity by 1.6% for 168 h at 80 °C, while [PAPy][OH] and [PAHM][OH] showed a loss of conductivity by 4.8% and 7.9%. The ion exchange capacity (IEC) values of three AEMs in the 1 M NaOH solution at 80 °C were almost unchanged after the test of 168 h and were equal to 0.93 mequiv·g^−1^ for [PAPy][OH], 1.12 mequiv·g^−1^ for [PAPi][OH], and 1.03 mequiv·g^−1^ for [PAHM][OH]. The spirocyclic [ASD]+ cation and corresponding polymeric membranes exhibited the highest chemical stability in the alkaline solution at elevated temperatures.

The results suggest that alkaline stability studies on small-molecule model cations may provide a basis for evaluating the properties of the corresponding polymer membranes and have also inspired a feasible approach for the preparation of spirocyclic QA- based AEMs with improved long-term stability and performance.

There has been a period of exceptional progress in recent years concerning the development of several alkaline-stable AEMs that have remarkable AEM-FC stability. Certain AEMs based on cycloaliphatic quaternary ammonium (cQA) (mainly five- and six-membered) appear to be among those ones, having the most promising performance overall.

**Scheme 6:**
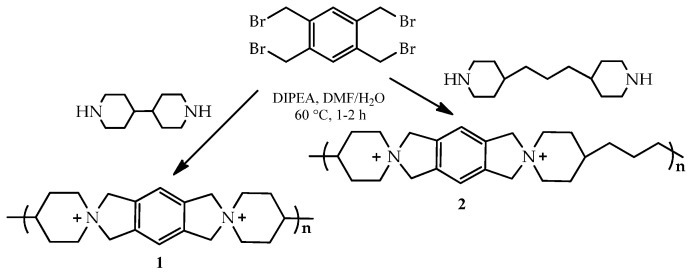
Cyclopolycondensations of tetrakis(bromomethyl)benzene with 4,4′-bipiperidine and 4,4′-trimethylenedipiperidine to form the spiro-ionene **1** and **2**. Reprinted with permission from [135]. Copyright 2017 American Chemical Society.

The development of cationic polymers for anion exchange membranes (AEMs) with high alkaline stability and conductivity is a considerable challenge in materials chemistry [135]. Two spiro-ionenes (**1**,**2**) from the commercially available building blocks have been obtained. Thus, tetrakis(bromomethyl)benzene (4BMB) has been employed in straightforward cyclopolycondensations with two commercially available dipiperidines, 4,4′-bipiperidine (BP) and 4,4′-trimethylenedipiperidine (TMDP), to form spiro-ionene **1** and **2**, respectively. Spiro-centered QA cations are formed directly in the polymer backbone (Scheme 6).

These polyelectrolytes show excellent thermal and alkaline stability, with no degradation detected when using NMR spectroscopy after more than 1800 h in 1 M KOD/D_2_O at 80 °C. Transparent and mechanically robust AEMs based on the ionically cross-linked blends of spiro-ionene and polybenzimidazole reach OH^−^ conductivities of up to 0.12 S cm^−1^ at 90 °C. The current findings demonstrate that spiro-ionenes constitute a new class of alkali-stable anion-exchange polymers and membranes.

**Figure 8:**
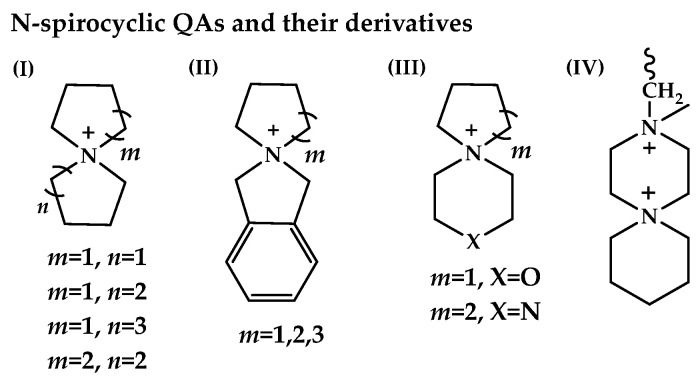
The representative cQAs, reported in the AEM literature: cN-spirocyclic QAs and their derivatives: (**I**) azoniaspiro-[m,n]-alkanes QAs; (**II**) benzo-N-spirocyclic QAs; (**III**) N-spirocyclic QAs with additional heteroatoms; (**IV**) bis-quaternized 3,6-diazaspiro-[5.5]-undecane. Reproduced with permission from [136]. Copyright 2022 Springer Nature.

The stability of anion exchange membranes (AEMs) is a long-standing challenge, limiting the widespread development and adoption of AEM fuel cells (AEM-FCs). Certain AEMs based on cycloaliphatic quaternary ammonium (cQA) (mainly five- and six-membered) appear to be among those that have the most promising overall performance (Figure 8) [136].

**Scheme 7:**
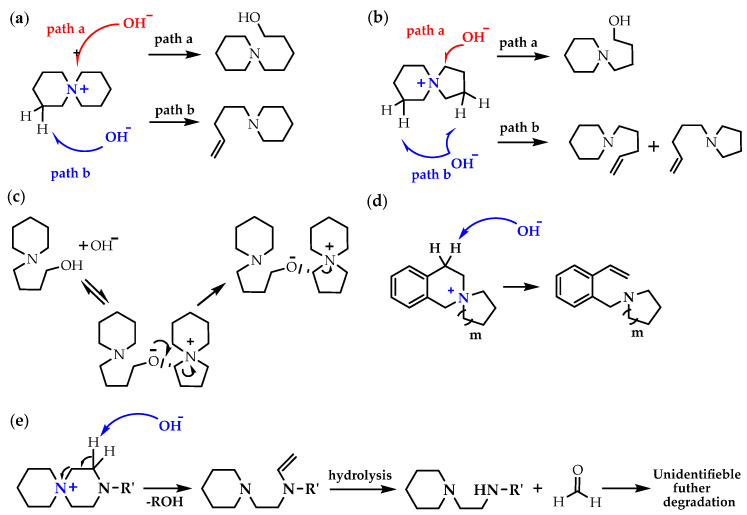
(**a**) The degradation mechanism for the ASU: the nucleophilic substitution (path a) and Hofmann elimination (path b); (**b**) the degradation mechanism for the ASD: the nucleophilic substitution (path a) and Hofmann elimination (path b); (**c**) the proposed cross-linking mechanism of the ASD from the nucleophilic substitution products; (**d**) the dominant degradation mechanism for the benzo-ASD: nucleophilic substitution; (**e**) the proposed major degradation mechanism for spirocyclic piperiziniums. Reproduced with permission from [136]. Copyright 2022 Springer Nature.

N-Spirocyclic QAs (generalized as azoniaspiro-[m,n]-alkanes QAs), benzo-N-spirocyclic QAs, N-spirocyclic QAs with additional heteroatoms (i.e., O,N), and bis-quaternized 3,6-diazaspiro-[5.5]-undecane have been utilized as components for AEMs. Usually, the degradation mechanisms for different types of cQAs are nucleophilic substitution (path a) [137] and Hofmann elimination, leading to alkenes (path b) [Scheme 7a,b] [138,139,140]. However, depending on the chemical environment of the central nitrogen and structural arrangement, one degradation pathway can dominate over another; in addition, products formed along one of the paths a or b can undergo subsequent transformations [Scheme 7c–e].

Cyclic QAs have proven to be exceptionally alkaline stable at temperatures up to 160 °C and NaOH concentrations up to 10 mol L^−1^, with piperidine-based 6-azonia-spiro[5.5]undecane having the highest half-life (110 h) under the chosen conditions [141]. The high alkaline stability of the six-membered azoniaspiro ring cation is attributed to the constrained ring conformation, which raises the transition state energy of both the elimination and substitution degradations.

The development of different anion exchange membranes (AEMs) for use in alkaline fuel cells and water electrolyzers based on the polystyrene (PS)-carrying benzyltrimethyl ammonium cations has been carried out [142].

**Scheme 8:**
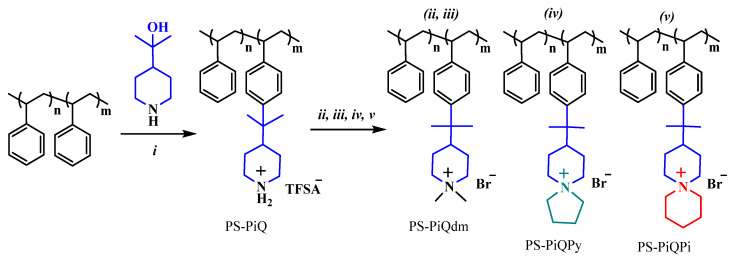
The synthetic route to PS-PiQdm, PS-PiQPy, and PS-PiQPi via the Friedel−Crafts alkylation, followed by different quaternization reactions. Reprinted with permission from [142]. Copyright 2016 American Chemical Society. Reaction conditions: (***i***) triflic acid, dichloromethane, 0 °C; (***ii***) iodomethane, K_2_CO_3_, N-methyl-2-pyrrolidone (NMP), r.t.; (***iii***) ion exchange in 1 M NaBr, 50 °C; (***iv***) 1,4-dibromobutane, DIPEA, NMP, 80 °C; (***v***) 1,5-dibromopentane, DIPEA, NMP, 80 °C.

Highly alkali-stable mono- and spirocyclic piperidine-based cations have been implemented in polystyrene (PS)-carrying benzyltrimethyl ammonium cations by first performing superacid-mediated Friedel–Crafts alkylation using 2-(piperidine-4-yl)propane-2-ol. This is followed by the quaternization of the piperidine rings, either using iodomethane to produce N,N-dimethyl piperidinium cations (PS-PiQdm) or by cycloquaternizations, using 1,5-dibromopentane and 1,4-dibromobutane, respectively, to obtain N-spirocyclic quaternary ammonium cations (PS-PiQPy and PS-PiQPi). Thus, it is possible to functionalize up to 27% of the styrene units with the piperidine rings, subsequently achieving complete quaternization (Scheme 8).

The synthetic approach ensures that all of the sensitive β-hydrogens of the cations presented in the piperidine ring, which are attached to the polymer polystyrene (PS) backbone in the Friedel–Crafts reaction step, are protected from an attack by hydroxyl ions OH^−^ by the two methyl groups.

The AEMs based on these polymers show high alkaline stability, and less than 5% ionic loss was observed when using ^1^H NMR spectroscopy after 30 days in 2 M aq NaOH at 90 °C. The AEMs functionalized with N,N-dimethyl piperidinium cations (PS-PiQdm) show higher stability than the ones carrying the N-spirocyclic quaternary ammonium (PS-PiQPy, and PS-PiQPi). A careful analysis of the latter revealed that the rings formed in cyclo-quaternization are more prone to degrade via Hofmann elimination than the rings, introduced in the Friedel–Crafts reaction. AEMs with an ion-exchange capacity of 1.5 mequiv·g^−1^ reach a hydroxide conductivity of 106 mS cm^−1^ at 80 °C under fully hydrated conditions. AEMs are further tuned and improved by blending with polybenzimidazole (PBI). For example, an AEM containing 2 wt % PBI shows a reduced water uptake and much improved robustness during handling and reaches 71 mS cm^−1^ at 80 °C. The study demonstrates that the critical alkaline stability of PS-containing AEMs can be significantly enhanced by replacing benchmark benzyltrimethyl ammonium cations [143] with N-alicyclic piperidine-based cations. However, the stability of these state-of-the-art cations needs to be further improved.

The alkaline stability of AEMs is greatly affected by the chemical structure of the membranes, including their polymer backbones, grafted side chains, and cationic functional groups. Commercialized aromatic polymers, such as polysulfones, poly(2,6-dimethyl-1,4-phenylene oxide)s, polyketones, etc., have been widely investigated for use as the backbones of AEMs; however, polyaromatics usually contain hetero atoms like O and S, which will lead to alkaline instability for both the backbones and the cationic groups [144].

In addition to polymer backbones, the grafted side chains of AEMs also play a critical role in chemical stability. Conventional benzylic quaternary ammonium-functionalized chains have proven to be sensitive to nucleophilic attacks, degrading the neighboring ether links in aromatic polymer backbones [145].

Currently, very few studies on N-spirocyclic quaternary ammonium-functionalized anion exchange membranes have been reported. Some researchers have prepared N-spirocyclic quaternary ammonium cations containing polymer backbones; however, their performance still needs to be improved. Data relating to their tensile strength and elongation at break are absent in most of the membranes, which might be related to brittle membranes caused by highly rigid N-spirocyclic main chains [146,147].

N-spirocyclic quaternary ammonium cations have been introduced into grafted side chains, extending the application scope of N-spirocyclic groups to many commercialized high-performance polymers. However, the conductivity of the as-prepared membranes has been poor due to the rigid piperidine spacer of the N-spirocyclic side chains [148].

5-Azonia-spiro-[4.5]-decane (ASD) and 6-azonia-spiro-[5.5]-undecane (ASU) cations have been grafted as functional groups to investigate the effects of ring size on the properties of the membranes [149]. Both ASD- and ASU-based AEMs exhibit excellent alkaline stability (around 95% retention of conductivity after the immersion in 1 M KOH solution at 80 °C for 720 h), high conductivity (up to 85.7 mS cm^−1^ at 80 °C), and feasible tensile strength and elongation at break (Table 3).

The performance of AEMs containing ether-spaced N-spirocyclic proposed in this work is among the best reported for N-spirocyclic functionalized AEMs in the literature, showing a better balance between conductivity and mechanical stability.

### 2.5. The Use of Metal-Free Small Organic Molecules as Phase-Transfer Catalysts

#### 2.5.1. The State of the Art

In the course of organic synthesis, many waste products can form in addition to the desired products since the transformations of the synthesis intermediates are not quantitative and selective processes. These waste products should be regenerated, destroyed, and disposed, which consumes significant energy and places a heavy burden on the environment. Therefore, it is of great importance to develop and use synthetic methodologies that minimize these problems. One of the most general and efficient methodologies that fulfill this requirement is phase-transfer catalysis (PTC).

This methodology is applicable to a wide variety of reactions in which inorganic and organic anions react with organic substrates. The methodology consists of the use of a heterogeneous two-phase system, in which one phase is a reservoir of reacting anions or a base for generating the organic anions, while the organic reagents and catalysts are located in the second organic phase. The reacting anions are continuously introduced into the organic phase in the form of ion pairs of lipophilic anions with lipophilic cations supplied by the catalyst [150]. In organic synthesis, it is often necessary to carry out reactions between water-soluble and oil-soluble reagents. Starks found that organic soluble quaternary ammonium or phosphonium cations, Q^+^, are suitable agents for the transport of anions from the aqueous phase to the organic phase. They act as powerful reaction accelerators [151].

Phase-transfer catalysts allow a reactant to migrate from one phase to another where the reaction takes place, eliminating the need for costly and unsafe solvents that can dissolve all reactants in one phase and costly raw materials, minimizing the waste issue. 

Phase-transfer catalysts are an alternative or complement to the existing, more traditional biocatalyzed and transition metal-catalyzed systems, representing a highly attractive method for the preparation of complex and multifunctionalized organic molecules [152]. The development of metal-free small organic molecules as catalysts has been a landmark advancement in organic synthesis in the recent past [153]. Asymmetric phase-transfer catalysis is based on the use of structurally well-defined chiral, non-racemic catalysts [154].

**Scheme 9:**
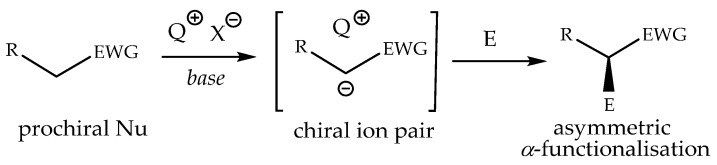
Generally accepted ion-pairing mechanism between the chiral cation Q^+^ of the PTC and an enolate and subsequent asymmetric α-functionalization with an electrophile (E). EWG = an electron-withdrawing group [155].

Chiral phase transfer or ion pair catalysis has become one of the fundamental catalytic principles due to the fact that it enables a wide range of powerful asymmetric reactions. One of the main advantages of all these chiral cations (Q^+^X^−^) phase-transfer catalysts is their unique ability to control the reactivity of a wide range of different prochiral nucleophiles through the formation of chiral ion pairs, which then undergo stereoselective α-functionalization reactions with various electrophiles (Scheme 9) [155].

Over the last two decades, tremendous efforts have focused on the design of novel high-performance organocatalysts to realize unprecedented reactions, including asymmetric transformations. In this context, the rational design of various types of chiral phase-transfer catalysts and their successful application to a wide variety of asymmetric transformations is considered to have the potential to create privileged structures [156,157].

In recent decades, chiral phase-transfer catalysts containing proton donor groups have become an extremely important strategy in the development of new catalysts; a large number of enantioselective reactions have been developed using PTC with proton-donating groups (OH, phenol, amide, and urea) [158].

It is worth noting that in addition to chiral cation-based phase-transfer catalysts (like the aforementioned onium and binaphtyl-based catalysts), the use of chiral anion-based PTCs has recently also attracted a lot of attention and led to a variety of highly versatile methods for use in asymmetric catalysis. In contrast to chiral cation-based PTCs, which mainly operate through the coordination and control of the nucleophile, these anionic PTCs usually coordinate cationic (and often hardly soluble) electrophilic reagents. This complementary strategy has also been rather impressively used for different asymmetric heterofunctionalization reactions [159,160].

**Figure 9:**
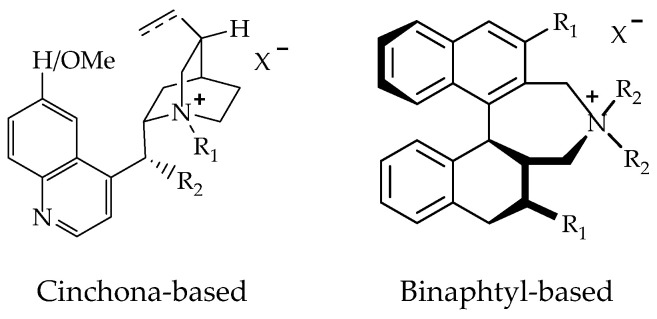
The most popular phase transfer catalyst of the chiral quaternary ammonium salt type [161].

«Alfa Chemistry Catalysts» is specialized in terms of providing various categories of catalysts, including chiral catalysts, ligands, metal catalysts, non-metal catalysts, phase-transfer catalysts, and photocatalysts. Catalysts are widely used in 90% of all commercially produced chemical products. Commonly used phase-transfer catalysts are chiral onium salt and chiral crown ethers. The phase-transfer catalyst of chiral onium salts may be a chiral quaternary ammonium salt or a chiral quaternary phosphonium salt. The phase-transfer catalyst of the chiral quaternary ammonium salt type is currently the most mature type of phase-transfer catalyst. Among them, cinchona-based skeletons and binaphthyl skeletons have achieved great success in terms of asymmetric transformation (Figure 9) [161].

#### 2.5.2. Azoniaspiro Compounds as Phase-Transfer Catalysts

Chiral PTCs are employed in various asymmetric syntheses like the enantioselective alkylation, epoxidation and Darzens condensation, Michael addition, asymmetric 1,6-conjugate addition, and others [162].

- *Catalytic asymmetric alkylation reaction*

**Scheme 10:**
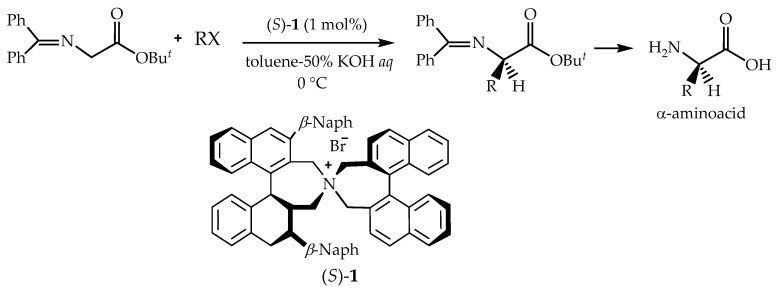
Synthesis of α-amino acids. Reproduced from [163]. Copyright 2000 Pergamon.

C2-symmetric chiral quaternary ammonium salt (S)-1 serves as a chiral phase-transfer catalyst in the catalytic enantioselective alkylation of tert-butyl glycinate-benzophenone Schiff base under mild phase-transfer conditions (Scheme 10) [163].

(S)-1 catalysts type can be readily assembled in the same manner; starting from either (R)- or (S)-binaphthol, a wide variety of natural and unnatural α-amino acids can be synthesized in an enantiomerically pure form via phase-transfer catalytic alkylation.

- *The Darzen reaction and epoxy condensation*

**Figure 10:**
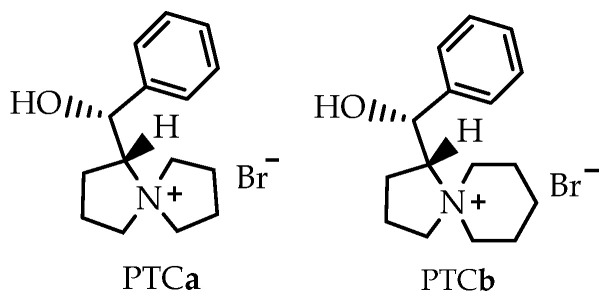
New PTCs: (R)-1-((R)-hydroxy(phenyl)methyl)- 5-azaspiro [4.4] nonan-5-ium bromide (PTC**a**) and (R)-1-((R)-hydroxy(phenyl)methyl)- 5-azaspiro [4,5] decan-5-ium bromide (PTC**b**). Reproduced from [164]. Copyright 2019 SNCSC.

The synthesis of new phase-transfer catalysts (PTCs) has been described. These catalysts were synthesized from proline with competitive yields. They were designed and synthesized in such a way that out of the four sides, three sides of quaternary nitrogen are blocked with cyclic groups and one aromatic group at α- or β-position to the nitrogen, whereas only one side is available for ion pairing with an anion [164]. These PTCs {(R)-1-((R)-hydroxy(phenyl)methyl)-5-azaspiro-[4.4]-nonan-5-ium bromide (**a**) and (R)-1-((R)-hydroxy(phenyl)methyl)-5-azaspiro-[4,5]-decan-5-ium bromide (**b**)} have the OH group at the β-position to the quaternary nitrogen, which can help lead to hydrogen bonding or attractive van der Waals interaction, which are advantageous for enhancing enantioselectivity (Figure 10).

**Scheme 11:**
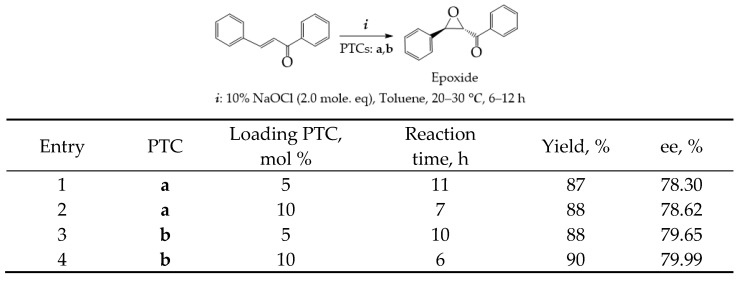
Epoxidation of chalcone to get a chiral epoxide by using the novel chiral PTCs (**a**, **b**). Reproduced from [164]. Copyright 2019 SNCSC.

**Scheme 12:**
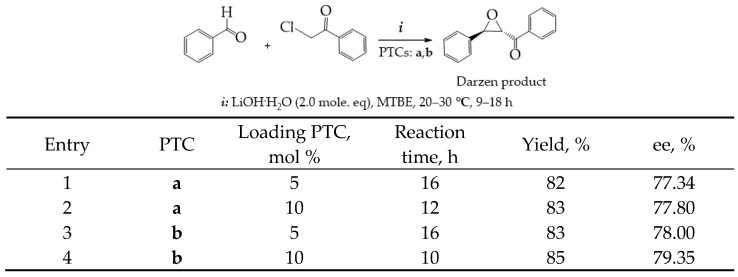
The Darzen condensation to get a chiral epoxide by using the novel chiral PTCs (**a**, **b**). Reproduced from [164]. Copyright 2019 SNCSC.

The effectiveness of these novel chiral cyclic phase-transfer catalysts has been evaluated by applying them in the enantioselective epoxide synthesis from α,β-unsaturated ketone (chalcone) and Darzens condensation (Scheme 11 and Scheme 12).

In the first case, the reaction was carried out in a two-phase system consisting of a 10% solution of NaOCl and toluene at a temperature close to r.t. (Scheme 11); in the second case, mild synthesis conditions were also used in a two-phase system consisting of an aqueous solution of LiOH·H_2_O (2.0 mol. eq) and methyl tert-butyl ether (MTBE) at a temperature close to r.t. (20−30 °C) (Scheme 12).

The obtained results show that the synthesized derivatives of proline are as effective as PTCs since the reaction products have been obtained with high yields (87–90% and 82–85%) and excellent enantioselectivity (78.30–79.99 and 77.34–79.35%), respectively.

**Scheme 13:**
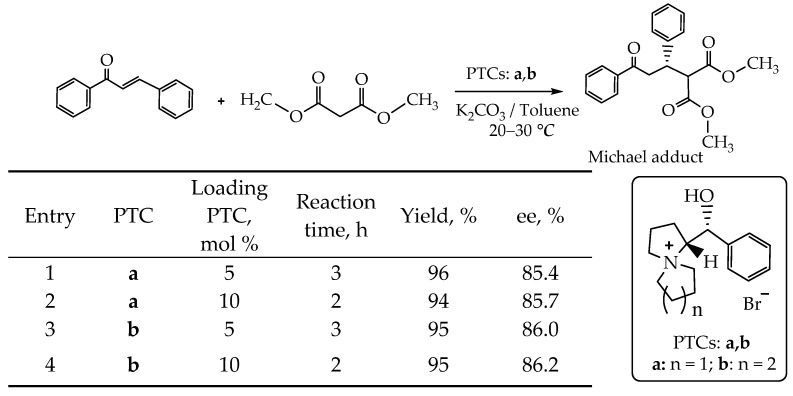
The Michael addition of dimethyl malonate to benzalacetophenone by using the chiral cyclic PTCs (**a**, **b**) to get the Michael adduct dimethyl-(*S*)-2-(3-oxo-1,3-diphenylpropyl)malonate. Reproduced from [165]. Copyright 2019 SNCSC.

- *The Michael addition*

(A) A Michael addition reaction with the formation of a carbon–carbon bond through the use of a catalytical methodology can provide enantiomerically pure Michael adducts. An example of Michael addition is the enantioselective addition of malonic esters to α,β-unsaturated carbonyls, which results in optically active tricarbonyl Michael adducts. The reaction, as shown in Scheme 13, uses the PTCs **a**,**b**, as discussed in the previous reference [165]. 

**Scheme 14:**
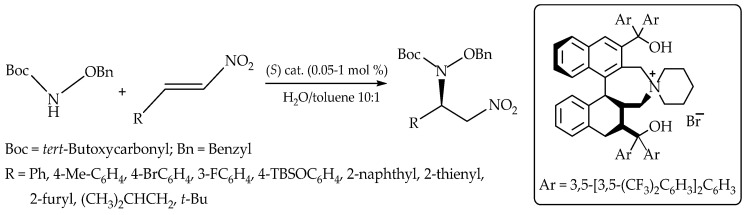
The asymmetric aza-Michael addition reaction catalyzed by the phase-transfer catalyst [166].

Phase transfer reactions are mostly carried out in two or three-phase systems in which generally aqueous and nonpolar solvent mixtures are used. Here, to obtain a chiral Michael adduct, a simple and easy-to-implement solid–liquid biphasic system was used. All of the reaction compounds, including chiral PTCs, were loaded into toluene and stirred at a temperature close to r.t. The reaction mixture was filtered after the completion of the reaction, and the filtrate was concentrated under a vacuum to obtain a crude product as a residue, which was purified via recrystallization from methanol to obtain pure Michael adducts. It has been observed that PTCs **a** and **b** produce good enantioselectivity at 86%; at the same time, the yield of the products was high, amounting to 95%.

(B) The asymmetric aza–Michael reaction (aza-MR) is an important synthetic tool for forming new C–N bond(s) for the development of new libraries of bioactive nitrogen compounds. The asymmetric conjugate amination of *tert*-butylbenzyloxycarbamate to β-nitrostyrene in the presence of chiral bi-functional tetraalkylammonium bromide as the chiral PTC was performed in a water-rich biphasic solvent. The reaction proceeded with high ee values of up to 95%, and very good yields (≈99%) were achieved in all cases (Scheme 14) [166].

- *The catalytic asymmetric 1,6-conjugate addition*

**Scheme 15:**
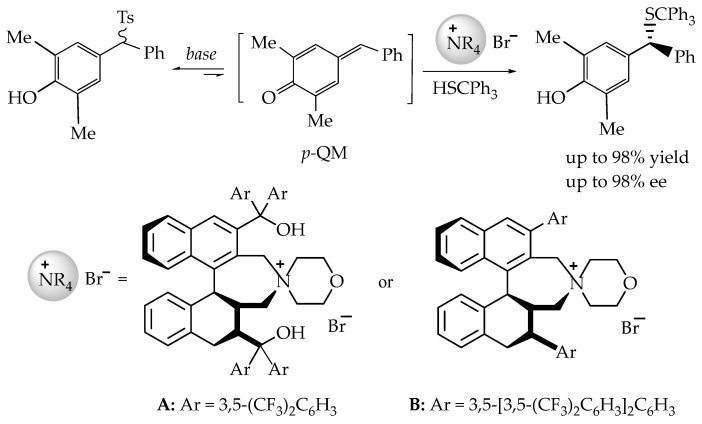
Catalytic behavior of the chiral binaphthyl OH modified catalyst **A** and chiral binaphthyl catalyst **B** in the reaction of catalytic asymmetric 1,6-conjugate addition. Reproduced from [167]. Copyright 2018 Royal Society of Chemistry.

A binaphthyl-based phase-transfer catalyst has been used in the reaction of asymmetric 1,6-conjugate addition to in situ generated *para*-quinone methides (*p*-QM), with 2,6-dialkyl-4-[phenyl(tosyl)methyl]phenol as substrates and tritylthiol as a nucleophile (Scheme 15) [167].

*p*-QM [168] has strong dipolar characteristics and a thermodynamic driving force that promotes rearomatization. This reaction provides a convenient route to optically active α-substituted benzylthioethers, forming the basis of the structures of bioactive molecules [169].

Using 2,6-dimethyl derivatives as an example, the conditions for the PTC reaction have been worked out. The reaction proceeds smoothly under the conditions developed by the authors in the presence of a saturated aqueous solution of Na_2_CO_3_ in CH_2_Cl_2_ at r.t. It has been found that quaternary ammonium salts, such as tetrabutylammonium bromide (TBAB), could smoothly promote the reaction of 2,6-methyl-4-[phenyl(tosyl)methyl]phenol with tritylthiol (Table 4, entry 1).

A comparative study of the catalytic behavior of various chiral quaternary ammonium salts has shown that tetrabutylammonium bromide (TBAB) smoothly promotes the reaction of 2,6-dimethyl-4-[phenyl(tosyl)methyl]phenol with tritylthiol, yielding the product with a quantity of 90% but in a racemic form. A study of the catalytic behavior of bifunctional chiral quaternary ammonium salts **A** revealed an improvement in yield (98%) and enantioselectivity (90% ee, entry 2) compared to TBAB. However, the reaction with chiral ammonium salt **B** leads to a high product yield that exists in the racemic form (90%, entry 3).

It has been found that the best solvent for catalyst **A** is methyl *tert*-butyl ether (MTBE), which provides optimal performance in terms of yield and enentioselectivity (entries 5–7). Finally, a gram-scale reaction was carried out to evaluate the synthetic potential of the process. High enantioselectivity persists with a slight decrease in catalyst yield (entry 7). The generality of this reaction was further investigated using optimal conditions found by examining 17 structurally diverse starting samples of 2,6-disubstituted-4-[phenyl(tosyl)methyl]phenol. It is noteworthy that the desired products were obtained in excellent quantities with high enantioselectivity in a short time at r.t. using equivalent amounts of reagents at a 1 mol% loading of bifunctional chiral catalyst **A**.

The influx of different novel recyclable phase-transfer catalysts is essential to overcome the large consumption, environmental pollution, difficulty in recycling, and high toxicity associated with traditional phase-transfer catalysts [157].

### 2.6. Organic Compounds as Structure-Directing Agents in Zeolite Synthesis

#### 2.6.1. The State of the Art

Due to their exceptional catalytic and selective adsorption properties in combination with thermal and chemical stability, zeolites have enormous use in oil refining and petrochemistry, with an increasing number of applications in fine chemical synthesis and environmental catalysis. Zeolites have come to dominate the world catalyst global market [170,171].

The consideration of zeolites as a future material is due to the many facile synthesis methods that can be used to obtain different structures with variations in pore size, surface area, pore volume, and physical properties. Every year, there is an increase in the number of new zeolite structures; their database, created by the International Zeolite Association (IZA), contains more than 250 known topologies [172].

Synthetic zeolites are typically obtained via the crystallization of gels containing water, silica, and alumina sources (and other metals: B, Ga, Ge, and so forth) under hydrothermal conditions at basic pH with temperatures in the range 60–200 °C. Usually, the gel includes cations of the structure-directing agents (SDAs). SDAs could be alkali metal cations (Na^+^, K^+^, Li^+^, etc.) or positively charged organic molecules and mineralizing agents (OH^−^ or F^−^). The crystallization mechanism of zeolites is usually described by a classical combination of nucleation and crystal growth steps [173].

The use of the organic SDAs is associated with the concept of the host–guest chemistry of organic molecules and inorganic systems during zeolite synthesis [174]. The addition of these organic molecules to the zeolite reaction mixtures provokes a particular ordering of the inorganic units around them, which directs the crystallization pathway towards a unique zeolite framework; hence, they are called structure-directing agents. In recent years, thanks to the use of SDAs in the synthesis of zeolites, tremendous progress has been achieved; the discovery of an extremely large number of new zeolite frameworks has taken place. Several properties determine the structure-directing effect of these organic species, especially their molecular size and shape, hydrophobicity, rigidity vs. flexibility, and hydrothermal stability [175]. SDAs were first used as pore generators in the synthesis of high-silica zeolites but also produced an effect on the crystallization evolution of zeolite, the size of the crystal, and zeolite chemical composition [176].

The latest research attempts to transfer the asymmetric nature of organic chiral molecules used as structure-directing agents to the zeolite lattice to produce the chiral enantioselective frameworks, which is one of the biggest demands in the current material chemistry [177].

Organic SDAs must meet appropriate criteria for hydrophobicity/hydrophilicity due to the C/N+ ratio, which is typically between 11 and 16, which limits the number of organic SDA candidates that can be used.

Zeolites may be classified according to their pore size as microporous (a diameter less than 2 nm), mesoporous (a diameter between 2 and 50 nm), and macroporous (up to 50 nm), as well as based on their surface area and physical properties [178]. There is also a parallel classification of the pore size of zeolites based on the membership of rings formed by the primary structural units of zeolites—tetrahedra (TO_4_) from SiO_4_^−4^ and AlO_4_^−5^. Therefore, we have the following: eight-member rings, which are often referred to as small-pore-size zeolite; ten-member rings, which are referred to as medium-pore-size zeolite; and twelve-member rings, which are considered large-pore-size zeolite [179].

The global SDAs market is estimated to grow by 7.6% and reach USD 1167.04 million by 2033. The growth of the SDAs market is due to the growing demand for zeolite in many industries, including chemical, oil and gas processing, and many others [180].

#### 2.6.2. Azoniaspiro Compounds as Structure-Directing Agents

**Scheme 16:**
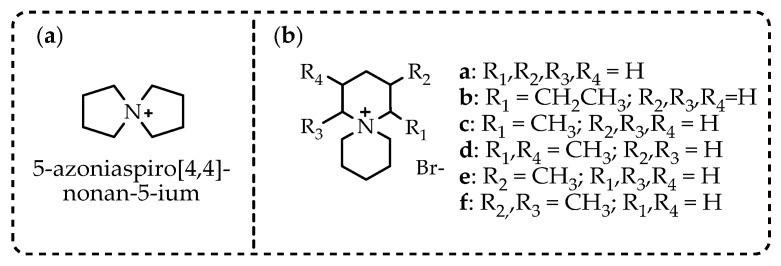
Development of azoniaspiro compounds as the SDAs. Reproduced from [181,182]. Copyright 2016 Elsevier.

Azoniaspiro compounds have found applications as structural-directing agents in the synthesis of zeolites. The hydrothermal syntheses of zeolites have been carried out using the azoniaspiro compound—5-azoniaspiro-[4,4]-nonane as an SDA in a fluoride-free medium. Germanosilicate zeolites attract great attention due to the fact that a number of extra-large pore systems with ring sizes exceeding 12-membered rings have been reported. A phase-pure IWW-type zeolite was obtained after hydrothermal treatment at 175 °C for 3 days with an Si/Ge ratio of around 4.9 [Scheme 16a] [181].

When researching the use of azoniaspiro compounds as SDAs, the Millini R. group focused their attention on substituted 6-azonia-spiro-[5,5]-undecanes; five different mono- and di-substituted azoniaspiro compounds **b**–**f** were obtained and used in the hydrothermal syntheses of zeolite (Scheme 16). Compounds **b**–**f** demonstrated relatively low selectivity; various topologies of zeolite structures were obtained: MTW, MOR, Beta, ERS-10, and defect-free MEL. MOR was obtained with all SDAs, and its formation has been favored by a low SiO_2_/Al_2_O_3_ molar ratio in the reaction mixture. One of the azoniaspiro compounds—(l-ethyl-6-azonia-spiro-[5,5]-undecane yielded another interesting result: the crystallization of pure, defect-free zeolite MEL 9 [Scheme 16b] [182].

**Scheme 17:**
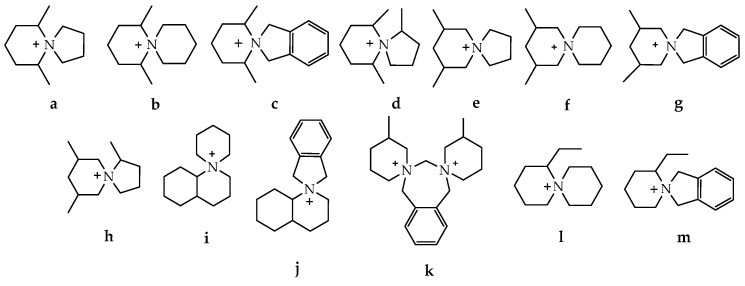
The hindered azoniaspiro compounds (**a**–**m**) in the synthesis of the UTL extra-large-pore germanosilicates. Reproduced from [183]. Copyright 2010 American Chemical Society.

Extra-large-pore germanosilicates with a UTL topology have been synthesized in basic media using a wide variety of sterically loaded spiroazonia compounds (**a**–**m**) as the SDAs. The composition of the reaction mixture and nature of the SDAs (structure, hydrophilicity/hydrophobicity balance, rigidity, and pKa) influenced phase selectivity and the degree of crystallinity in the UTL structure (Scheme 17) [183].

The optimum synthesis time was considered to be 3–7 days at a Si/Ge ratio of 2 and (Si + Ge)/SDA molar ratios of 1.7–6. Zeolite with UTL structure shows very high thermal stability; the structure does not collapse after calcination, even at 1000 °C. The micropore volume of the best crystals is 0.21–0.23 cm^3^/g, and the micropore diameter is ~1.0 nm.

In this work, the following selection criteria for organic molecules for use as potential SDAs have been used:

(1) SDA organic molecule should contain one or two quaternary N atoms and have a C/N ratio in the range of 10–16. (2) An SDA molecule should possess sufficient rigidity. It is desirable that its structure should include two or more five- or six-membered aliphatic hydrocarbon rings. The spiro-structure, especially with a charged N atom in place of a joining of two rings, as well as the presence of benzene rings, is useful. (3) An SDA molecule should possess optimum hydrophobicity (log P = −1.8 and −0.6). (4) An SDA should possess sufficiently high stability under the hydrothermal synthesis of zeolites. (5) The kinetic diameter of the SDA molecule should be within 7–15 Å.

It has been stressed that zeolites have a more perfect structure and higher micropore volume when they are crystallized from the reaction mixtures, having higher SDA template contents. In contrast, when using the large SDA molecules (SDA**c**, SDA**g**, SDA**j**, and SDA**k**), it is necessary to decrease the template contents (Si + Ge)/SDA.

## 3. Conclusions

Based on the fact that 2-aminospiropyrazolinium compounds and structurally related azoniaspiro compounds belong, in a broad sense, to the class of ionic liquids, we have reviewed them and studied their practical applications. In general, the concept of ionic liquids existing in a molten state at temperatures below 100 °C and close to r.t. as non-volatile, non-flammable, and recyclable materials is very attractive, and these materials are used in many technological applications. However, there are applications, such as phase-transfer catalysts, structure-directing agents, pharmaceutical additives to improve the dissolution of poorly soluble drugs, etc., where ionic organic compounds which are not liquid at r.t. can be used. Such properties of well-studied azoniaspiro compounds would give impetus to the investigation of similar properties of the little-studied spiropyrazolylammonium compounds, which we have considered only in relation to their in vitro antitubercular, antidiabetic, and antimicrobial activity.

## 4. Future Directions

Using design approach and based on the available 2-aminospiropyrazolinium cations when selecting anions, there is a possibility of synthesizing new compounds with useful properties. At the same time, it is necessary to address the issues of the stability of such new compounds. It must be taken into account that durability with the preservation of the structure is a function of a long period of time, certain temperature limits, and specific environments. In addition, environmental problems remain, such as the disposal of deteriorated ILs and the study of the toxic properties of ILs.

## Figures and Tables

**Table 1:** The comparative illustration of the practical constraints of A-WE, PEM-WE, and AEM-WE [127].

Parameters	A-WE	PEM-WE	AEM-WE
Separation	Diaphragm	PEM	AEM
Cathode	Nickelmolybdenum alloys	Pt group metals	Transition metals
Anode	Nickelcobalt alloys	RuOx, IrOx	Transition metals
Current collector plate	Nickel	Copper	Copper
Bipolar plate	—	Graphite and Ti	Ni or SS
Electrolyte	KOH	Pure water	Alkali solution, pure water
Current density	<0.5 A cm^−2^	1–2 A cm^−2^	1–2 A cm^−2^
Operating temperature	60–90 °C	50–90 °C	40–80 °C
Gas purity	>99.5%	>99.99%	>99.99%
Lifetime	~100 kh	<10 kh	<2 kh
Estimated cost	Low	High	—
Technology status	Mature	Commercial for small scale	Research and Development

**Table 2:** Common anion-conducting cations in the AEM head group [131].

Nitrogen-Containing Groups	Nitrogen-Free Groups
Quaternary ammonium/tertiary diamines	Phosphonium
(Benz)Imidazolium	Sulphonium
Guanidinium	Metal cations (Ruthenium, Nickel, Cobalt)
Pyridinium	

**Table 3:** The AEMs with the N-spirocyclic quaternary ammonium groups. Reproduced from [149]. Copyright 2020 Elsevier.

AEMs	Structure/Cation	IEC (mmol·g^−1^)	WU(%)	SR(%)	HC(mS·cm^−1^)	Ts (MPa)	Eb (%)	Stabil./ 80 °C
			80 °C					
Cr-ASD-PSF 2.05	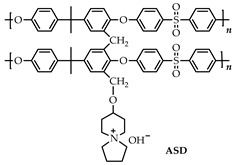	2.05	92.7	39.6	85.7	10.5	12.8	94.9% (1 M KOH, 720 h)
Cr-ASU-PSF 1.92	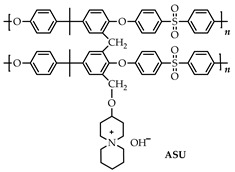	1.92	91.1	37.7	78.8	8.3	17.8	95.6% (1 M KOH, 720 h)

Abbreviations: PSF: polysulfone; Cr: crosslinked; IEC: ion exchange capacity; WU: water uptake; SR: swelling ratio; HC: hydroxide conductivity; Ts: tensile strength; Eb: elongation at break. Stability: percentage of remaining hydroxide conductivity, the test conditions, comprising the concentrations of the alkaline solution, temperature.

**Table 4:** The reaction conditions to determine the catalytic activity of the catalysts **A** and **B**. Reproduced from [167]. Copyright 2018 Royal Society of Chemistry.

Entry	Chiral PTC	Solvent	Time, h	Yield, %	ee, %
1	5 mol%, TBAB	CH_2_Cl_2_	2.0	90	0
2	5 mol%, **A**	CH_2_Cl_2_	0.5	98	+90
3	5 mol%, **B**	CH_2_Cl_2_	1.5	90	0
4	5 mol%, **A**	toluene	0.5	90	+97
5	5 mol%, **A**	MTBE	0.5	98	+98
6	1 mol%, **A**	MTBE	3.0	98	+96
7	1 mol%, **A**	MTBE	5.0	95	+96

## Data Availability

Not applicable.

## Abbreviations

ILs	ionic liquids
DIPEA	diisopropylethylamine
MTB H37Rv	drug-sensitive strains of *M. tuberculosis*
MTB MDR	Multidrug resistant strains of *M. tuberculosis*
GC	Green Chemistry
TSCA	Toxic Substances Control Act
r.t.	room temperature
TSCA	Toxic Substances Control Act
m.p.	melting point
[emim]Cl^−^AlCl_3_	1-ethyl-3-methylimidazolium chloride aluminum chloride
HF	hydrogen fluoride
FSI	bis(fluorosulfonyl)imide
[PF_6_]	hexafluorophosphate
Emim-Cl	1-ethyl-3-methylimidazolium chloride
Bmim-Cl	1-butyl-3-methylimidazolium chloride
Bmpy-Cl	1-butyl-1-methylpyrrolidinium chloride
NBuPy-Cl	n-butylpyridinium chloride
LOELs	lowest observed effect levels
[Ch][AA] ILs	cholinium amino acid ionic liquids
[Ch][Ac]	cholinium acetate
Td	degradation temperature
EC_50_	the concentration of ionic liquids at which the growth of microorganisms and cells is halved compared to that without the ionic liquid
QA	quaternary ammonium
LOELs	lowest observed effect levels
T_onset_	thermal stability of ionic liquids—decomposition temperature, °C
FP	flash point
TGA/DSC	thermogravimetry and differential scanning calorimetry
TGA-FTIR	thermogravimetric analysis coupled with Fourier transformed infrared spectrometry
AA ILs	сholinium and amino acid-based ionic liquids
[Ch][AcO]	choline acetate
[Ch][Cl]	choline chloride
[Ch][For]	choline formate
[Ch][Lac]	choline lactate
[Ch][Pro]	choline propionate
[Ch][Ole]	choline oleate
BHET	bis-hydroxyethyl terephthalate
CHILs	carbohydrate-based ILs
MWCNTs	multiwalled carbon nanotubes
GTA	N-[2-(d-glucopyranosyl)ethyl]-N,N,N-trimethylammonium) cations
[N(SO_2_CF_3_)_2_]^−^	bis((trifluoromethyl)sulfonyl)methanide
QA BF_4_	quaternary ammonium tetrafluoro borate
UNFCCC	United Nations Framework Convention on Climate Change
GHG	greenhouse gas
FCHEA	Fuel Cell and Hydrogen Energy Association
A-WE	alkaline water electrolysis
AEM	anion exchange membrane
AEM-WE	anion exchange membrane water electrolysis
PEM-WE	proton exchange membrane water electrolysis
SO-WE	solid oxide water electrolysis
AEM-FCs	anion exchange membrane fuel cells
PEM-FCs	proton exchange membrane fuel cells
ASD	5-azonia-spiro-[4.5]-decane
ASU	6-azonia-spiro-[5.5]-undecane
PTC	phase-transfer catalysis
*p*-QM	*para*-quinone methides
TBAB	tetrabutylammonium bromide
MTBE	methyl *tert*-butyl ether
IZA	International Zeolite Association
SDAs	structure-directing agents
